# High-throughput chemical and chemoenzymatic approaches to saccharide-coated magnetic nanoparticles for MRI†

**DOI:** 10.1039/c9na00376b

**Published:** 2019-07-29

**Authors:** Thomas W. Fallows, Andrew J. McGrath, Joana Silva, Simon G. McAdams, Andrea Marchesi, Floriana Tuna, Sabine L. Flitsch, Richard D. Tilley, Simon J. Webb

**Affiliations:** School of Chemistry, University of Manchester Oxford Road Manchester M13 9PL UK S.Webb@manchester.ac.uk +44 (0)161 306 4524; Manchester Institute of Biotechnology, University of Manchester 131 Princess St Manchester M1 7DN UK; School of Chemistry, University of New South Wales Australia; Australian Centre for NanoMedicine, University of New South Wales Australia; School of Materials, University of Manchester Oxford Road Manchester UK; Photon Science Institute, University of Manchester Oxford Road Manchester M13 9PL UK; Electron Microscope Unit, Mark Wainwright Analytical Centre, University of New South Wales Australia

## Abstract

There is a need for biofunctionalised magnetic nanoparticles for many biomedical applications, including MRI contrast agents that have a range of surface properties and functional groups. A library of eleven adducts, each formed by condensing a reducing sugar with a catechol hydrazide, for nanoparticle functionalisation has been created using a high-throughput chemical synthesis methodology. The enzymatic transformation of an *N*-acetylglucosamine (GlcNAc) adduct into an *N*-acetyllactosamine adduct by β-1,4-galactosyltransferase illustrates how chemoenzymatic methods could provide adducts bearing complex and expensive glycans. Superparamagnetic iron oxide nanoparticles (8 nm diameter, characterised by TEM, DLS and SQUID) were coated with these adducts and the magnetic resonance imaging (MRI) properties of GlcNAc-labelled nanoparticles were determined. This straightforward approach can produce a range of MRI contrast agents with a variety of biofunctionalised surfaces.

## Abstract 2

Magnetic nanoparticles coated with a glycan-catechol adduct, selected from a library created through a simple condensation reaction and an enzyme-catalysed reaction, were assessed as MRI contrast agents.

## Graphical abstract

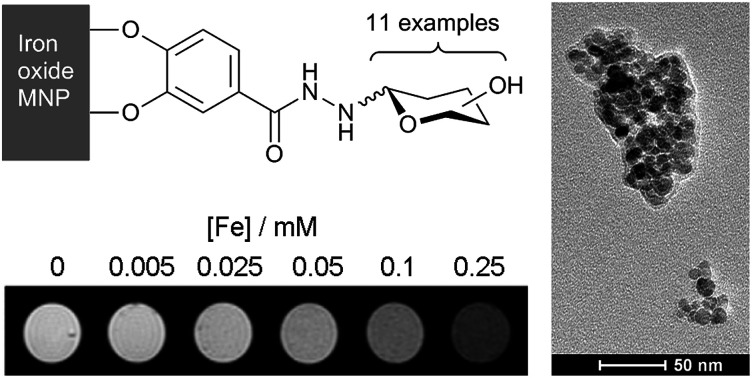

## Introduction

Cell targeting magnetic nanoparticles (MNPs) with the ability to bind to specific cell types have many current and potential applications, including in magnetic resonance imaging (MRI),^1^ magnetic hyperthermia,^2,3^ and magnetic separations.^4,5^ A popular current approach towards cell targeting is use of antibody-coated nanoparticles, but several issues exist with this approach, particularly cost.^6^ Cell–cell recognition processes suggest an alternative approach, as cell adhesion is often mediated through recognition of an ensemble of cell surface glycans (oligosaccharides).^7^ This produces highly specific but tuneable recognition of different cell types, which has made the surface glycosylation of nanoparticles of particular interest.^8^ If libraries of saccharide-terminated ligands for MNPs were available, then nanoparticle surfaces could be tailored post synthesis using different coating ligands that are mixed *in situ*, allowing optimised binding to the suite of cell surface lectins on a targeted cell line.

One interesting application of cell-targeting saccharide-coated MNPs would be *in vivo* labelling agents for MRI. MRI is a well-established diagnostic tool for imaging tissues within the body^9^ that circumvents the use of contrast agents with short half-lives (as in positron emission tomography)^10^ and offering better resolution at greater depths than is achievable by ultrasound.^11^ MRI contrast agents increase differences in the *T*_1_ (spin–lattice) and *T*_2_ (spin–spin) relaxivity of water protons^1^ in different tissues, allowing better imaging of internal organs. Gd^3+^ complexes are currently the most commonly used MRI contrast agents and are primarily useful for *T*_1_-weighted images, but Gd^3+^ leeching in the body has been suggested as a cause of nephrogenic systemic fibrosis.^12^ Superparamagnetic iron oxide nanoparticles (SPIONs) are an alternative that act as *T*_2_ contrast agents.^13^ SPIONs can be surface functionalised (for example with catechol derivatives) to improve stability, to increase circulation time, to add therapeutic agents, or to introduce targeting groups.^14^ Beyond labelling tissues for MRI, biofunctionalised MNPs might be useful for magnetic biosensing,^15^ where an *in situ* binding process gives a change in magnetic signal. For example, it has been shown that using lectins to aggregate saccharide-coated nanoparticles leads to a measureable decrease in the *T*_2_ value.^16^ It may be possible to image similar aggregation processes *in vivo*, perhaps allowing the real-time tracking of biochemical processes on MNP surfaces or MNP agglomeration on targeted cancerous cells in the early stages of tumour development.

**Fig. 1:**
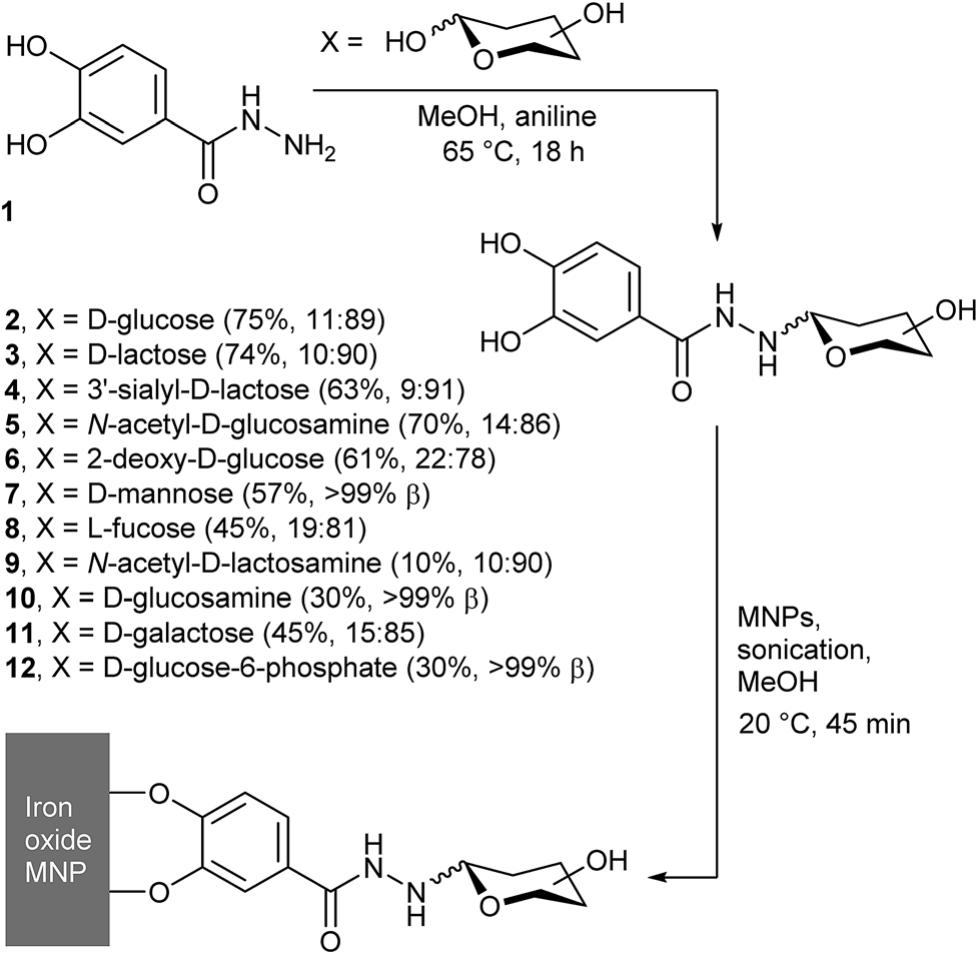
General scheme showing condensation of 3,4-dihydroxybenzhydrazide with reducing sugars, to give conjugates 2–12. In brackets after each compound is the yield and the α : β anomeric ratio.

We have recently shown that condensing catechol hydrazide 1 with four different reducing sugars gave the corresponding saccharide–catechol conjugates (2 to 5, Fig. 1).^17^ Purification of the crude mixtures by high-performance liquid chromatography (HPLC) gave the four adducts in a high-throughput process, with costs commensurate with that of the starting saccharide. These adducts were coated onto the surface of MNPs, to give saccharide-coated MNPs designed to exploit the affinity of cell-surface lectins for specific saccharide motifs. These coatings were found to be quite stable; hydrolysis of the hydrazone link in the resorcinol analogues is slow at pH 7.4 (*ca.* 25% after 24 days) and desorption of the catechol group from a MNP surface is even slower (*t*_1/2_ for dissociation >8 weeks).^17^ Both cell-surface and lectin recognition of these coated MNPs has been shown,^17^ with cell targeting by glucose (Glc) and *N*-acetylglucosamine (GlcNAc) labelled MNPs used to impart magnetophoretic behaviour on 3T3 fibroblasts.^18^

To grow these four published examples into a library, we have explored the reactivity of another nine reducing saccharides with catechol hydrazide 1. We then explored the potential of chemoenzymatic synthesis to expand this library and include more complex and expensive saccharides. SPIONs were coated with these saccharide–catechol conjugates and the potential of saccharide-coated SPIONs as MRI contrast agents was explored, which included the effect of adding a conjugate lectin.

## Results and discussion

### Chemical synthesis of saccharide adducts 2–12

We previously reported that the aniline-catalysed reaction of 3,4-dihydroxybenzhydrazide 1 with glucose, *N*-acetylglucosamine, lactose, and 3′-sialyllactose gives the corresponding hydrazones, which cyclise to form the pyranoside adducts.^17^ The same simple, high-throughput method (Fig. 1) was now applied with d-sugars 2-deoxyglucose, mannose, *N*-acetyllactosamine (LacNAc), glucosamine, galactose, glucose-6-phosphate, Lewis^X^ trisaccharide and Lewis^X^ tetrasaccharide, as well as l-fucose.

In addition to aniline, other catalysts for hydrazone and oxime formation were tested, such as those assessed by Wendeler *et al.*^19^ and Crisalli *et al.*^20^*p*-Phenylenediamine and anthranilic acid were chosen due to their combination of high reported catalytic activity and relatively low cost. Reactions between 1 and glucose in NMR tubes were carried out in CD_3_OD at 65 °C for each catalyst, as well as a control with no catalyst present. ^1^H NMR spectra were measured at 0, 1, 2, 4, 6, 8 and 24 h and the yields were calculated by integrating the anomeric peaks of glucose and the glucose adduct 2 (see the ESI†). These studies showed that an overnight reaction gave the optimal balance between reaction time and yield, as rapid equilibration of the glucose anomers was followed by a steady increase in the fraction of adduct 2 formed over the 24 h. This reached 20% for the uncatalysed reaction, but both *p*-phenylenediamine and anthranilic acid gave *in situ* conversions in excess of 80% (see the ESI†). These conversions were better than that of aniline (55%), but after reversed-phase HPLC purification an aniline catalyst was found to give the purest product; this catalyst was favoured thereafter.

Purification of the products 6–12 by reversed-phase HPLC separation was most effective if multiple aliquots (0.5 mL) were separated on a semi-preparative column; a single crude reaction mixture (*ca.* 60 mg) could usually be purified during one day. The collected fractions were then concentrated under reduced pressure to remove the organic solvent, before being lyophilised to give the products as white powders. The products were analysed by ^1^H NMR spectroscopy, which showed no resonances from open chain hydrazone (typically found at 6.5–8 ppm)^21^ but resonances in the 3.7–4.7 ppm region from the anomeric protons of ring closed pyranosides. This region of the spectra showed that our HPLC conditions did not separate the α- and β-anomers of the adducts. However, integration of the resonances provided the α : β ratio (see the ESI†) in the purified mixtures, which showed the β-anomer was favoured, from 78% for 6 to >99% for 7, 10 and 12 (Fig. 1).

Under these reaction conditions, the yields for the condensation of 1 with uncharged saccharides were fair overall (generally 40 to 70%), except in the case of *N*-acetyllactosamine, which only gave a low yield of the adduct (yield = (10 ± 2)%). More complex adducts, such as those with Lewis^X^ trisaccharide and Lewis^X^ tetrasaccharide, could not be obtained in useful quantities due to low conversions combined with the high cost of the starting sugars. The yields in these cases were no greater than 13% and 35% respectively, although the very small amounts obtained (0.7 and 1.2 mg respectively) precluded full characterisation. Analysis of the by-products of these reactions also provided evidence of fragmentation of the oligosaccharides under the reaction conditions. In particular, the fucose adduct 8 (identified by ^1^H NMR spectroscopy) was isolated from the crude mixture of Lewis^X^ tetrasaccharide in 10% yield (0.2 mg).

### Enzymatic synthesis of saccharide adduct 9

Given the low yield for LacNAc adduct 9, chemoenzymatic methods for synthesising this compound were explored. Glycosyltransferases give excellent regio- and stereochemical control, and several glycosyltransferases have been shown to act on unnatural substrates, including substrates immobilised on surfaces.^22^ β-1,4-Galactosyltransferase 1 (β4Gal-T1) has been shown to be particularly versatile in this respect, for example adding galactose to *N*-acetylglucosamines displayed on gold surfaces,^23^ glass surfaces,^24^ and lipid bilayers^25,26^ to give LacNAc labelled surfaces. Should GlcNAc adduct 5 be a substrate for β4Gal-T1, there is also the potential for this transformation to be performed *in situ* on the nanoparticle surface.

**Fig. 2:**
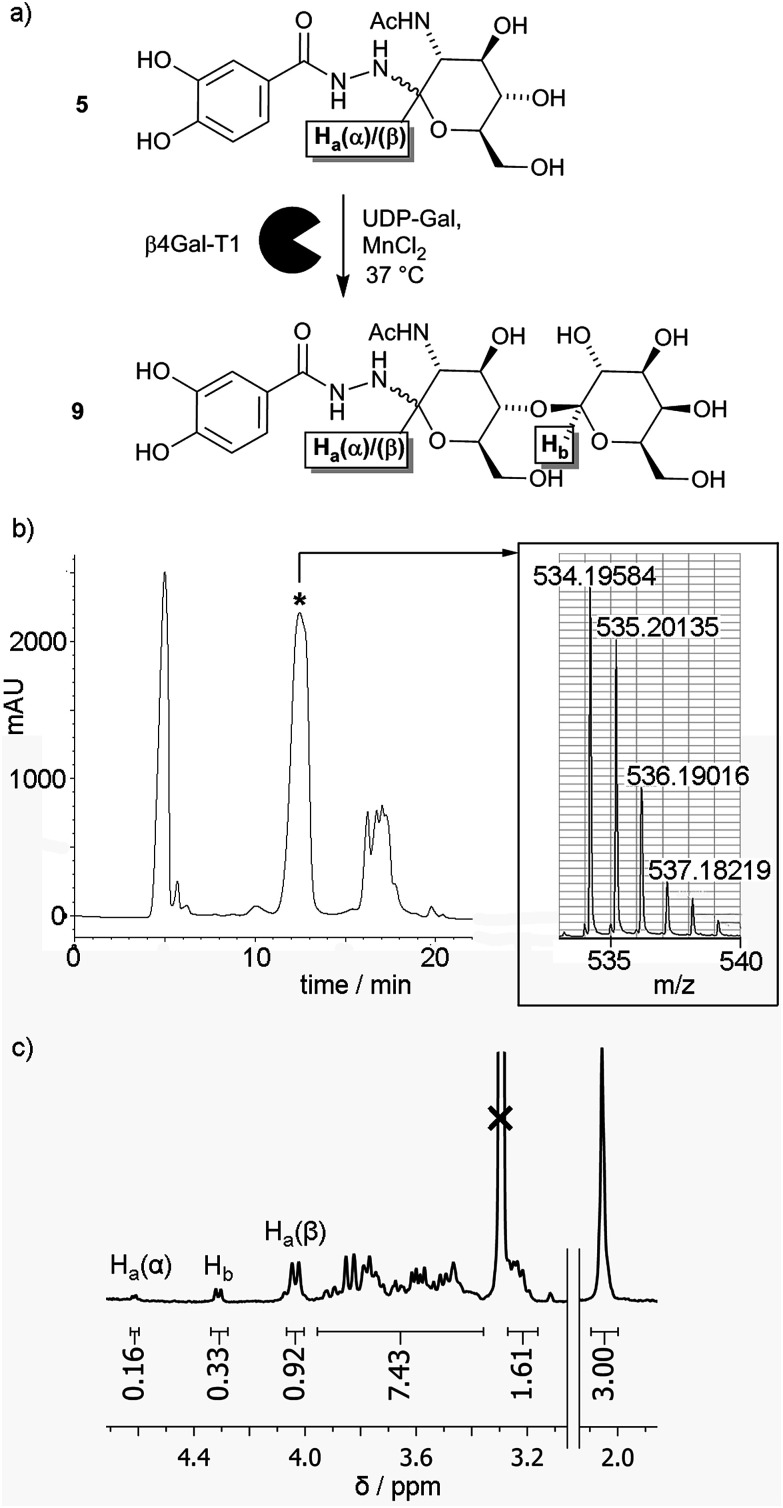
(a) Enzymatic transformation of GlcNAc adduct 5 into LacNAc adduct 9. (b) HPLC trace for the enzymatic transformation of 5 into 9, with the product containing fraction indicated (*). Inset: MS data indicating the enzymatic formation of 9. (c) Partial ^1^H NMR spectrum for the enzymatically produced mixture of 5 and 9, showing integrations of the anomeric proton resonances (as labelled in (a) above) relative to the integration of the methyl resonance (3H, shown right).

To assess if β4Gal-T1 could transform GlcNAc adduct 5 into LacNAc adduct 9, conditions that had been employed previously on synthetic GlcNAc glycolipids were applied (Fig. 2a).^25,26^ The GlcNAc adduct was dissolved in MES buffer (1 mL) along with β4Gal-T1 enzyme (14.75 μL of a 0.54 mg mL^−1^ solution), uridine diphosphogalactose disodium salt (UDP-Gal, 11.25 mg, 20 μmol) and MnCl_2_ (3 μL of a 1 M solution in water). The mixture was incubated at 37 °C overnight. After incubation, the reaction mixture was analysed by positive ion LCMS, which showed two ions with *m*/*z* slightly lower than that expected for the LacNAc product; [9 + H − 2H]^+^ and [9 + Na − 2H]^+^. The loss of two hydrogens was hypothesized to be due to Mn(ii)-catalysed aerial oxidation of the catechol moiety in 5, as the solution turned light brown after MnCl_2_ addition but remained colourless in the absence of MnCl_2_. Mixing 1 with MnCl_2_ in MES buffer in air gave the same colour change, but degassing of the solution followed by purging with argon before MnCl_2_ addition prevented this oxidation.

Repetition of the enzymatic transformation using samples under an Ar atmosphere, followed by LCMS, revealed that ions corresponding to the LacNAc product, [9 + H]^+^ and [9 + Na]^+^, were found in a mixed fraction that eluted at 12 minutes (Fig. 2b). Further attempts to purify this mixed fraction of 5 and 9 using other HPLC conditions still resulted in co-elution of 5 and 9. To determine the extent of the enzymatic transformation, the fraction containing 5 and 9 was analysed by ^1^H NMR spectroscopy. The 3.7–4.7 ppm region contains resonances from the α and β anomeric protons (H_a_(α) and H_a_(β) respectively) on the glucosyl moieties of both 5 and 9, as well as the exclusively β anomeric proton (H_b_) on the galactosyl moiety of 9. The integrations of the anomeric protons H_a_(α), H_a_(β) and H_b_ in 5 and 9 were 0.16, 0.92 and 0.33, respectively (Fig. 2c), which suggests that the conversion of 5 to 9 was about 30% after 24 h. This value was significantly lower than the 40% conversion after 1 h reported for the conversion of *p*-nitrophenyl-GlcNAc to *p*-nitrophenyl-LacNAc using β4Gal-T1 and UDP-Gal, and more similar to conversions obtained for synthetic GlcNAc-capped glycolipids (10–30% after 6 h).^25^ The overall percentage conversion was superior to that obtained by the chemical synthesis of adduct 9, although pure 9 has not yet been obtained using our HPLC method.

### Synthesis of saccharide-coated MNPs

Both chemical^27^ and enzymatic routes^28^ for iron oxide MNP synthesis exist. The simplest chemical route to iron oxide MNPs is the co-precipitation method,^29^ where Fe^3+^ and Fe^2+^ are mixed in a 2 : 1 ratio at basic pH, and there are a number of published methods that give good size control.^30^ In the absence of oxygen, the iron oxide formed is magnetite (Fe_3_O_4_), but this can be readily oxidised on the surface^31^ and in bulk to maghemite (γ-Fe_2_O_3_).^32^

Iron oxide nanoparticles were synthesised by the co-precipitation method, and stored under N_2_ to prevent oxidation. To coat the MNPs, the appropriate saccharide–catechol adduct (10 mg) was added to MNPs (10 mg) in methanol (5 mL) and the suspension was probe sonicated for 45 min. The coated MNPs were then sedimented by centrifugation and the supernatant was removed with the aid of a permanent magnet, which held and avoided disturbance of the MNP pellet. The nanoparticles were washed with methanol to remove any unbound adduct before re-suspension by bath sonication. The process of sedimentation and washing was repeated twice with methanol and once with Milli-Q filtered water. The coated nanoparticles were finally suspended in Milli-Q filtered water and lyophilised for storage.^17^

### Characterisation of uncoated and coated MNPs

**Fig. 3:**
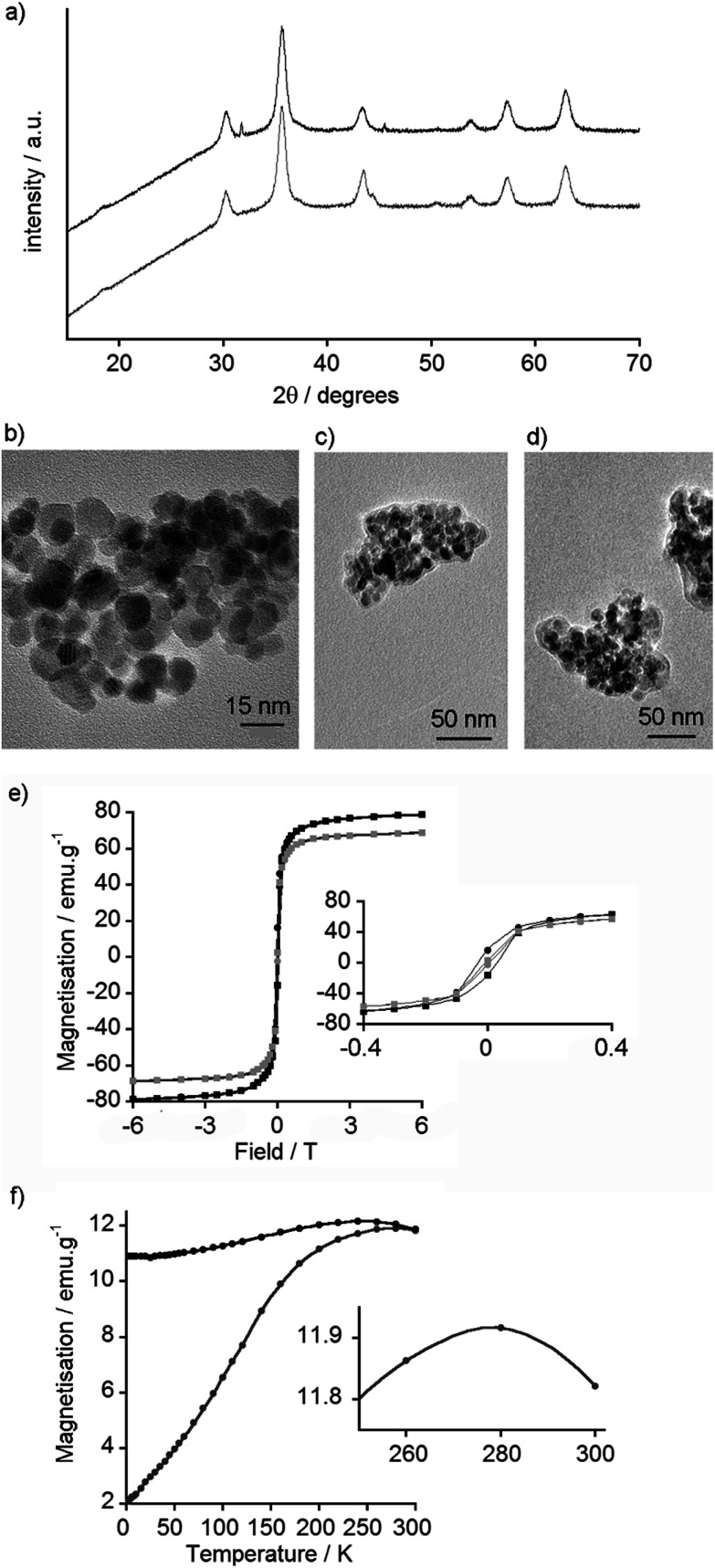
(a) XRD spectra of uncoated MNPs (black) and GlcNAc-coated MNPs (gray, offset). (b) TEM image of uncoated MNPs. (c) TEM image of GlcNAc-coated MNPs showing cluster size and carbonaceous coating corona. (d) TEM image of GlcNAc-coated MNPs with WGA (0.1 mg mL^−1^). (e) SQUID measurement of magnetisation *vs.* field curves for uncoated MNPs at 5 K (black) and 300 K (gray). Inset: Expansion to show hysteresis. (f) SQUID measurement of ZFC (gray) and FC (black) curves for uncoated MNPs. Inset: Expanded section of ZFC curve showing blocking temperature of 278 K.

The type of iron oxide in the MNPs can be inferred from the X-ray powder diffraction (XRD) pattern. This information can be further supplemented with high-resolution transmission electron microscopy (TEM), which not only provides a measure of particle diameter but may also show the crystalline structure of the iron oxide. XRD patterns obtained for MNPs coated with 2–12 were very similar to each other and to that of the uncoated nanoparticles (see the ESI†). For example, the XRD patterns of uncoated and GlcNAc-coated MNPs are similar (Fig. 3a) and comparable to both magnetite and maghemite. The XRD of maghemite, however, has weak peaks at 23.77° (210) and 26.10° (211)^33^ and there are no clear peaks in this 20–30° region in the XRD pattern of the MNPs, which may suggest these MNPs are magnetite. Nonetheless both magnetite and maghemite nanoparticles are known to exhibit superparamagnetism.^34^ Inductively Coupled Plasma Atomic Emission Spectroscopy (ICP-AES) was then used to estimate the extent of coating on the nanoparticle surface. Phosphorus can be quantified by ICP-AES, therefore MNPs coated with the (glucose-6-phosphate)–catechol adduct 12 were investigated. These coated MNPs had 1.64% w/w phosphorus, which shows 12 covers around 59% of the available surface area of each MNP (see the ESI†); this value is similar to that found for another catechol-based conjugate coated on MNPs.^35^

Nanoparticle size is an important factor for determining both biological responses to particles and their magnetic behaviour; the formation of single magnetic domains will give superparamagnetic behaviour. TEM of the synthesised MNPs showed that most particles were smaller than 20 nm in diameter (Fig. 3b), with an average size of 8.3 nm (standard deviation 2.4 nm, *n* = 100, see ESI†), which is in a range typical for particles formed by this method.^29,36^ Little difference was observed by TEM between coated and uncoated nanoparticles, and both appeared to agglomerate into small clusters around 200 nm in diameter. The GlcNAc-coated MNPs, however, showed a visible ‘corona’ around the nanoparticle clusters (Fig. 3c), something that is often suggestive of carbonaceous coatings.^37^ Interestingly, despite finding GlcNAc-coated MNPs bound to Quartz Crystal Microbalance with Dissipation monitoring (QCM-D) chips coated with the GlcNAc-recognising lectin wheat germ agglutinin (WGA),^17^ the addition of WGA to 5-coated MNPs did not appear to significantly change the extent of interparticle aggregation (Fig. 3d).

Dynamic light scattering (DLS) measurements were performed on uncoated MNPs and representative coated MNPs (coated with LacNAc conjugate 9, sialic acid conjugate 4 and the catechol hydrazide 1), which might be expected to provide coatings with neutral, anionic and cationic surface charges respectively. DLS indicated there was a significant amount of aggregation in Milli-Q water but the suspensions gave poor quality scattering data. Passing the suspensions through a 200 μm pore size filter resulted in better data, showing aggregates typically 80–200 nm in diameter (81 nm for uncoated and 153 nm for 4-coated MNPs), usually with larger populations also present (*ca.* >1000 nm diameter, see the ESI†). In addition, changing the DLS scattering angle revealed particles in a freshly sonicated 9-coated MNP suspension that had a diameter of 7 ± 2 nm, close to that observed by TEM. The zeta potential was also determined for uncoated MNPs as well as MNPs coated with 1, 4 and 9. Uncoated particles were found to have a slightly anionic surface (−15 ± 8 mV), consistent with previous reports.^38^ Coating the particles with 9 produced little change in zeta potential compared to uncoated MNPs (−13 ± 8 mV), but the MNPs became more negative (−35 ± 4 mV) after coating with 4 and more positive (−4 ± 1 mV) after coating with 1. The relatively low zeta potentials, which are reduced further in HEPES and PBS buffers (see ESI†), may contribute to the propensity of these particles to aggregate.^39^

In order to determine if the particles were superparamagnetic iron oxide nanoparticles (SPIONs), the magnetic properties of uncoated MNPs were assessed using a Superconducting Quantum Interference Device (SQUID). Magnetisation *vs.* field curves revealed a coercive field of 0.31 kOe at 5 K, indicative of ferromagnetic behaviour, and a magnetic saturation of 79 emu g^−1^ at 6 Tesla (Fig. 3e). At 300 K, no hysteresis was observed, confirming the nanoparticles as superparamagnetic at room temperature, with a magnetic saturation of 69 emu g^−1^ at 6 Tesla (Fig. 3e). As the primary use for these nanoparticles is envisioned to be *in vivo* biological imaging at *ca.* 310 K, the SQUID data suggested these particles could be useful MRI contrast agents.

To determine the blocking temperature, zero-field cooled (ZFC) and field cooled (FC) measurements were performed under a 100 Oe field. The maximum point of the ZFC curve provides an estimate of the blocking temperature (Fig. 3f).^40^ This maximum at 278 K is higher than blocking temperatures commonly reported for dispersed Fe_3_O_4_ and γ-Fe_2_O_3_ MNPs, which are usually below 150 K.^41^ Nonetheless higher temperatures have been reported, especially when particles have aggregated and there are significant interactions between the MNPs.^42^

### Magnetic resonance imaging (MRI) properties of GlcNAc-coated MNPs

Given the SQUID data for uncoated MNPs, and to assess the effect of a saccharide coating on the ability of the MNPs to act as contrast agents,^43^ the *T*_1_ and *T*_2_ relaxivity^44^ of GlcNAc-coated MNPs was determined. These measurements were performed in an MRI machine equipped with a 9.4 T magnet using a multi-slice-multi-echo sequence. Five MNP suspensions with total iron concentrations, as measured by ICP-AES, up to 0.5 mM in PBS (pH 7.4) were created. Agar (2 g) was dissolved in PBS (100 mL, pH 7.4) and heated using a microwave oven until it was fully dissolved. GlcNAc-coated MNP suspensions (1 mL) were then mixed together with agar solution (1 mL). The agar decreases inhomogeneity due to MNP sedimentation and aggregation, and after cooling and thickening the gels contained an even dispersion of embedded nanoparticles. In addition, the lectin WGA (100 μg of 36 kDa lectin, 5.6 nmol binding sites) was mixed with 5-coated MNP suspensions and fixed in agar in the same way. Recognition of 5-coated MNPs by surface-bound WGA has been demonstrated using QCM-D^17^ but the effect of lectin binding on the magnetic properties of MNPs was unknown, for example a change in the extent of aggregation could alter *T*_2_.^16^ Like many plant lectins, the lectin WGA has a relatively low affinity for its conjugate saccharides (*K*_d_ for GlcNAc of 760 μM).^45^ Although the concentration of GlcNAc on the MNPs (estimated as 13 μM at [Fe] = 0.25 mM, see the ESI†) and WGA binding site concentration (1.4 μM) are both lower than the *K*_d_, a cluster glycoside effect at the nanoparticle surface may strengthen recognition.^46^

**Fig. 4:**
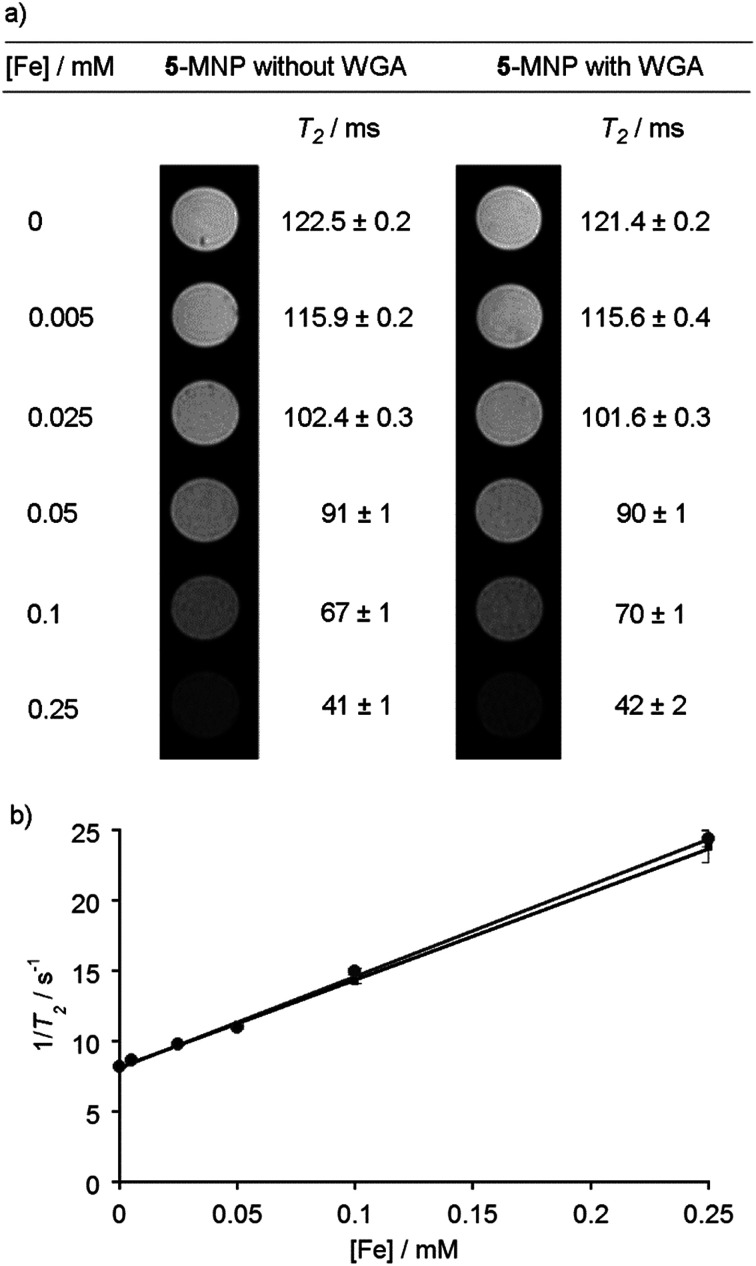
(a) MRI contrast images of GlcNAc-coated MNPs in agar at different concentrations, both with and without wheat germ agglutinin (WGA). Measured *T*_2_ values next to images. (b) Plot of iron concentration against 1/*T*_2_ for GlcNAc-coated MNPs, with (black squares) and without WGA (grey circles).

As the concentration of GlcNAc-coated MNPs increased, the spin–spin relaxation time, *T*_2_, decreased significantly, from 120 to 40 ms, both in the presence and absence of WGA. This is displayed visually as a darkening of the image, from the bright white of pure agar in PBS, to the dark grey image obtained with 0.25 mM [Fe] (Fig. 4a). The spin–lattice relaxation time, *T*_1_, was relatively unchanged for both samples, slightly decreasing from 3300 to 2700 ms as the MNP concentration increased.

The *r*_2_ value, which indicates the *T*_2_ relaxivity of an MRI contrast agent, can be calculated from the gradient of a plot of total iron concentration against 1/*T*_2_ (Fig. 4b).^47^ Without any WGA present, the *r*_2_ value was calculated to be (65 ± 8) mM^−1^ s^−1^, and in the presence of WGA the *r*_2_ value was (62 ± 8) mM^−1^ s^−1^. Both values compare well with other iron oxide MNPs of similar size formed by co-precipitation, and also with the values observed for previously-used clinical *T*_2_ MRI agents such as Ferumoxtran (60 mM^−1^ s^−1^).^44,48,49^ The corresponding *r*_1_ values were low (both (0.3 ± 0.2) mM^−1^ s^−1^, see the ESI†) providing *r*_2_/*r*_1_ ratios in the order of 200, which suggests that this class of saccharide-coated MNP would give viable contrast agents.^50^ Taken together, these data indicate that surface reaction of the MNPs with the adduct 5 has not significantly diminished the desirable magnetic properties of the MNPs. However, the addition of lectin did not change *r*_2_, which suggests that either insufficient lectin was bound to the MNP surface or the extent of MNP aggregation did not change sufficiently to produce a change in the relaxivity. The latter may be the case if the MNPs already have a tendency to agglomerate, as indicated by the DLS, TEM and SQUID data, whilst setting of the lectin/5-MNP mixtures in agar may have inhibited further aggregation.

## Conclusions

The aniline-catalysed condensation of reducing saccharides with commercially available catechol-hydrazide 1 has been shown to be a versatile method for providing magnetic nanoparticles with saccharide coatings. A library of eleven different mono-, di- and trisaccharide adducts has been developed, although the condensation methodology was ineffective for the more complex and expensive saccharides that were tested (Lewis^X^ trisaccharide and Lewis^X^ tetrasaccharide). The successful enzymatic transformation of GlcNAc-catechol adduct 5 into LacNAc-catechol adduct 9 suggests that chemoenzymatic methods could provide difficult-to-synthesise adducts, and give access to MNPs coated with complex oligosaccharides; synthetic GlcNAc derivatives have been elaborated into Lewis^X^ and Lewis^a^ through a series of enzymatic transformations.^51^ Although better HPLC methods for separating substrates from products should be developed, there is also the potential for these enzymatic transformations to be performed directly on adducts immobilised on the nanoparticle surface.

Iron oxide MNPs were synthesised by a co-precipitation method and shown to be 8 nm diameter SPIONs at room temperature (*T*_B_ ∼ 278 K). The blocking temperature was higher than that commonly observed for dispersed iron oxide MNPs, and was consistent with MNP agglomeration. The observation of 200 nm diameter aggregates of uncoated MNPs and GlcNAc-coated MNPs by TEM (in the presence and absence of the conjugate lectin) supported this proposition. TEM also showed that a GlcNAc coating did not produce significant morphological changes in individual MNPs. MRI measurements in agar showed that GlcNAc-coated particles had properties suitable as a MRI contrast agent, with shortening of the *T*_2_ relaxation times with increasing MNP concentration (*r*_2_ = (65 ± 8) mM^−1^ s^−1^) but little change in the *T*_1_ relaxation times. The addition of the conjugate lectin, WGA, did not significantly change these properties, perhaps because the size of the aggregates did not change significantly (as suggested by TEM data) or because the agar matrix inhibited further MNP aggregation. To provide saccharide coated MNPs that can detect lectins through aggregation-induced changes in magnetic properties, their intrinsic tendency to aggregate should be decreased, perhaps by increasing MNP surface charge (zeta potential) and/or the distance between the saccharide and the catechol surface anchor. Control over MNP aggregation will also be important for controlling MNP-cell interactions *in vivo*.^52^

The ability to rapidly obtain libraries of simple saccharide coatings using this high-throughput methodology should permit the creation of MNPs coated with mixtures of saccharides tailored to bind specific cell types. Preliminary data has shown that coating MNPs with 2 or 5 promotes recognition by 3T3 fibroblasts compared to uncoated MNPs, with indications that GlcNAc is better recognised than Glc by this cell line (although discrimination was relatively weak).^17,18^ Other cell lines may be more selective; we have shown HepG2 hepatocytes will take up LacNAc-coated lipid nanoparticles (liposomes) in preference to GlcNAc- and sialylLacNAc-coated liposomes, presumably through targeted binding to overexpressed asialoglycoprotein receptor on the surface of these liver cancer cells.^26^ Chemoenzymatic synthesis, as described here, of more complex coating adducts may also provide an additional level of cell selectivity. Selective recognition by non-adherent cells could provide applications in biomedical magnetophoresis,^53^ while *in vivo* MRI studies on animal models could confirm the performance of these MNPs as tissue contrast agents in a medically relevant context.

## Experimental

General synthesis reagents and WGA were purchased from Sigma-Aldrich Co. Ltd. (Dorset, UK). 3,4-Dihydroxybenzhydrazide was supplied by Fluorochem (Derbyshire, UK) and *N*-acetyllactosamine which was supplied by Carbosynth (Berkshire, UK). Permanent magnets were purchased from e-magnets UK, Hertfordshire, UK. Bovine β-1,4-galactosyltransferase 1 (β4Gal-T1) was obtained as previously described.^25^

Reversed-phase HPLC purification was performed on an Agilent 1100 series system with an Agilent Eclipse XDB-C18 (9.4 mm × 250 mm) column. NMR spectra were taken in deuterated solvents using a Bruker 400 MHz Avance spectrometer with broadband probe or a Bruker 800 MHz Avance III. NMR chemical shift values are referenced to residual peaks from non-deuterated solvent and measured in ppm. Multiples are reported as singlets (s), doublets (d), triplets (t), multiplets (m) or a combination of the above and coupling constants are measured in Hertz. Electrospray mass spectrometry was performed on a Micromass LCT instrument using a Waters 2790 separations module with electrospray ionization and TOF fragment detection. High resolution mass spectrometry was performed on a Water Q-TOF micro with an ES+/− ion source. Elemental analysis was performed using a Thermo Scientific FLASH 2000 series CHNS/O Analyser. Sonication of nanoparticles (*e.g.* for coating) was performed with a Sonics Vibra-Cell VCX 130PB Ultrasonic Processor (CV 188) with a stepped micro tip (3 mm × 136 mm) running at 130 W, 20 kHz and 50% amplitude. Bath sonication was carried out using a Camlab Transonic T460 operating at 35 kHz. Centrifugation was performed in 15 mL Falcon tubes using a Heraeus Megafuge 1.0R spinning at 4200 rpm at a constant temperature of 23 °C for 10 min.

### General procedure for the synthesis of adducts

Saccharide (0.3 mmol) and hydrazide (0.3 mmol) were dissolved in methanol with aniline (10 mL of 5 mM stock solution). The reaction was allowed to reflux overnight under a N_2_ atmosphere. After this time, the reaction was allowed to cool before removal of the solvent under reduced pressure. Purification was achieved by HPLC, with multiple aliquots (0.5 mL) of the reaction mixture separated on a semi-preparative column (9.4 mm × 250 mm) using a gradient method shifting linearly over 1 h (from 5% to 50% THF in water). The eluent was monitored by UV-visible spectroscopy (230 and/or 250 nm), and the uncharged saccharide–catechol adducts typically eluted between 14 and 18 min (1 mL min^−1^ flow rate). The product containing fractions were collected and freeze-dried to give the adducts as white powders.

### Chemoenzymatic synthesis of 9

Glycoconjugate 5 (10 μmol) was dissolved in 2-(*N*-morpholino)ethanesulfonic acid (MES) buffer (1 mL, 50 mM, pH 7.0) and mixed with UDP-Gal (20 μmol, 11.25 mg) and β4Gal-T1 (14.75 μL of a 0.54 mg mL^−1^ solution). After degassing the solution by sonication and purging it with argon, MnCl_2_ (3 μL of a 1.0 M solution in water) was added to the solution flask. The mixture was then vortex mixed followed by incubation overnight at 37 °C. The reaction mixture was directly purified by HPLC (Macherey-Nagel Nucleosil C18, 250 × 4.6 mm, 5 μm) using portion-wise (20–80 μL) addition to an analytical column and a 40 min gradient ranging from 5% to 100% acetonitrile in water (0.5 mL min^−1^ flow rate). The eluent was monitored by UV spectroscopy (260 nm) and the product containing fractions were collected and freeze-dried to afford a 7 : 3 mixture of compounds 5 and 9.

### General nanoparticle coating procedure

Iron oxide nanoparticles were synthesised by adding FeSO_4_·7H_2_O to Fe_2_(SO_4_)_3_·H_2_O in a 1 : 2 mole ratio in Milli-Q filtered water at 80 °C under a flow of N_2_. After vigorous stirring (15 min), NH_4_OH was added and stirred vigorously for a further 30 min. The resultant MNPs were washed with Milli-Q filtered water until a neutral pH was obtained, re-suspended in a NaCl solution (20 mL, 40 mM), lyophilised and stored under N_2_.

MNPs (10 mg) were suspended in methanol (5 mL) by probe sonication (130 W, 20 kHz, 50% amplitude) for 5 minutes. To this suspension was added the desired coating molecule (any of 2 to 12, 10 mg). The sample was sonicated with a probe sonicator for a further 45 min. Any unreacted coating material was removed by centrifugation (4200 rpm, 10 min) to give a pellet then supernatant removal (pellet held in place with aid of a permanent ring magnet, 0.51 T). The process of sedimentation and washing was repeated twice with methanol (2 × 10 mL) and once with Milli-Q filtered water until the coated nanoparticles were finally suspended in Milli-Q filtered water (3 mL) and either used immediately or lyophilised for storage.

### X-ray power diffraction (XRD)

XRD was performed at the University of Namur in the Namur Nanosafety Center/Namur Research Institute for Life Sciences. Diffraction patterns were obtained using a Philips PW3064 XPERT-PRO diffractometer using Cu Kα radiation. The MNP powders were placed over the aperture on a slide.

### Transmission electron microscopy (TEM)

TEM of uncoated nanoparticles was carried out using a Philips CM20 operated at 200 keV. Samples were prepared by suspending nanoparticles in methanol then dropping a dilute sample onto a carbon coated copper grid and leaving the solvent to evaporate. TEM of 5-MNPs in the absence and presence of lectin was carried out using a FEI Tecnai G2 20 operated at 200 keV. Samples were prepared by suspending the nanoparticles in water (1 mL) at a concentration of 0.01 mg mL^−1^. Half of the volume of each sample was removed and incubated with WGA (0.1 mg, 5.6 nmol binding sites) and incubated at room temperature for 4 h. The other half of each suspension of MNPs was left untreated. The samples were dispersed by vigorous shaking, drops of each were placed on TEM grids, and the solvent left to evaporate.

### Dynamic light scattering (DLS) and zeta potential measurements

DLS and zeta potential measurements (using the Smoluchowski equation) were carried out using a Malvern Zetasizer Nano ZSP 633 nm laser at 25 °C. Coated MNPs were suspended in PBS (pH 7.4), Milli-Q water or HEPES buffer (20 mM, pH 7.5 with 150 mM NaCl and 2 mM CaCl_2_) at concentrations of 6 × 10^−7^ mg mL^−1^. Particles were suspended by sonication with a probe-type sonicator for 10 min followed by filtration using a Minisart® syringe filter of 200 μm. Measurements were carried out in folded capillary zeta cells with a scattering angle of 13° or 173°.

### SQUID measurements of uncoated MNPs

Magnetic studies were carried out using a Quantum Design MPMS-XL SQUID magnetometer equipped with a 7 Tesla magnet. In order to prevent the samples from adopting an orientation in the applied magnetic field they were immobilised in an eicosane matrix and placed in gelatine capsules. Magnetic hysteresis data were recorded at 5 and 300 K, by cycling the magnetic field between (6 T) and (−6 T) fields (sequence used: (0) → (6 T) → (−6 T) → (6 T)). Zero field cooled (ZFC) and field cooled (FC) magnetisation data were collected under an applied static magnetic field of 100 Oe. For ZFC, the sample was initially cooled from 300 to 5 K at a rate of 10 K min^−1^ under zero-dc field: no data was collected at this step. After keeping the temperature stable at 5 K for 5 min, a small 100 Oe magnetic field was applied and ZFC data as a function of temperature was collected under a warming regime at 10 K min^−1^. For FC data, a field of 100 Oe was maintained when cooling the sample from 300 to 5 K without measurement, and then data were collected under same field upon warming from 5 to 300 K at 10 K min^−1^.

### Magnetic resonance imaging (MRI)

GlcNAc-coated MNPs were suspended in PBS (1 mL, pH 7.4, 10 mM) with Fe concentrations of 0.01 mM, 0.05 mM, 0.1 mM, 0.2 mM and 0.5 mM. For lectin binding, 5-MNPs (0.5 mM Fe concentration) were suspended in PBS (2 mL, pH 7.4, 10 mM), then WGA (100 μg, 5.56 nmol binding sites, in 50 μL Milli-Q filtered water) was added and the suspension incubated at room temperature for 3 h. Some of the sample was then diluted to make lower Fe concentrations of 0.01 mM, 0.05 mM, 0.1 mM and 0.2 mM. Control samples with and without lectin were also made without the addition of MNPs.

To prepare the samples for MRI, agar powder (2 g) was dissolved in PBS (100 mL, pH 7.4, 10 mM) by heating in a microwave oven for periods of approximately 40 s, with stirring in between, until fully dissolved. The MNP suspensions in PBS were added to an Eppendorf tube, and an equal amount of liquid agar solution added by pipette, followed by careful mixing by pipette (avoiding bubble formation). The tubes containing MNPs in agar were left to cool to room temperature, forming a solid gel containing dispersed MNPs.

MRI was performed using a Bruker BioSpec Avance III 94/20 Preclinical MRI. MR images were acquired at 9.4 T using a 2D multi-slice multi-echo sequence at 300 K for simultaneous *T*_1_ and *T*_2_ measurements.^37^ For MRI measurements, Eppendorf tubes containing MNPs in agar were inserted into a holder, and the holder inserted into the instrument.

## Conflicts of interest

There are no conflicts to declare.

## Supplementary Material

NA-001-C9NA00376B-s001

## References

[cit1] Stephen Z. R., Kievit F. M., Zhang M. (2011). Mater. Today.

[cit2] Chang D., Lim M., Goos J. A. C. M., Qiao R., Ng Y. Y., Mansfeld F. M., Jackson M., Davis T. P., Kavallaris M. (2018). Front. Pharmacol..

[cit3] Fourmy D., Carrey J., Gigoux V. (2015). Nanomedicine.

[cit4] Pamme N., Eijkel J. C. T., Manz A. (2006). J. Magn. Magn. Mater..

[cit5] Carr C., Espy M., Nath P., Martin S. L., Ward M. D., Martin J. (2009). J. Magn. Magn. Mater..

[cit6] Cheng Z., Al Zaki A., Hui J. Z., Muzykantov V. R., Tsourkas A. (2012). Science.

[cit7] Griffin M. E., Hsieh-Wilson L. C. (2016). Cell Chem. Biol..

[cit8] Sampaolesi S., Nicotra F., Russo L. (2019). Future Med. Chem..

[cit9] Zottis A. D. A., Beltrame J. M., Lara L. R. S., Costa T. G., Feldhaus M. J., Curi Pedrosa R., Ourique F., de Campos C. E. M., de A. Isoppo E., da Silva Miranda F., Szpoganicz B. (2015). Chem. Commun..

[cit10] Bass L. A., Wang M., Welch M. J., Anderson C. J. (2000). Bioconjugate Chem..

[cit11] Misri R., Meier D., Yung A. C., Kozlowski P., Hafeli U. O. (2012). Nanomedicine.

[cit12] Rogosnitzky M., Branch S. (2016). BioMetals.

[cit13] Gueron M. (1975). J. Magn. Reson..

[cit14] Hachani R., Lowdell M., Birchall M., Hervault A., Mertz D., Begin-Coline S., Thanh N. T. K. (2016). Nanoscale.

[cit15] Pankhurst Q. A., Connolly J., Jones S. K., Dobson J. (2003). J. Phys. D: Appl. Phys..

[cit16] Basuki J. S., Esser L., Duong H. T. T., Zhang Q., Wilson P., Whittaker M. R., Haddleton D. M., Boyer C., Davis T. P. (2014). Chem. Sci..

[cit17] Coxon T. P., Fallows T. W., Gough J. E., Webb S. J. (2015). Org. Biomol. Chem..

[cit18] Fallows T. W., Coxon T. P., Gough J. E., Webb S. J. (2017). MRS Adv..

[cit19] Wendeler M., Grinberg L., Wang X., Dawson P. E., Baca M. (2014). Bioconjugate Chem..

[cit20] Crisalli P., Kool E. T. (2013). J. Org. Chem..

[cit21] Baudendistel O. R., Wieland D. E., Schmidt M. S., Wittmann V. (2016). Chem.–Eur. J..

[cit22] Gray C. J., Weissenborn M. J., Eyers C. E., Flitsch S. L. (2013). Chem. Soc. Rev..

[cit23] Šardzík R., Green A. P., Laurent N., Both P., Fontana C., Voglmeir J., Weissenborn M. J., Haddoub R., Grassi P., Haslam S. M., Widmalm G., Flitsch S. L. (2012). J. Am. Chem. Soc..

[cit24] Serna S., Etxebarria J., Ruiz N., Martin-Lomas M., Reichardt N. C. (2010). Chem.–Eur. J..

[cit25] Noble G. T., Craven F. L., Voglmeir J., Šardzík R., Flitsch S. L., Webb S. J. (2012). J. Am. Chem. Soc..

[cit26] Craven F. L., Silva J., Segarra-Maset M. D., Huang K., Both P., Gough J. E., Flitsch S. L., Webb S. J. (2018). Chem. Commun..

[cit27] McGrath A. J., Cheong S., Henning A. M., Gooding J. J., Tilley R. D. (2017). Chem. Commun..

[cit28] Rawlings A. E., Bramble J. P., Walker R., Bain J., Galloway J. M., Staniland S. S. (2014). Proc. Natl. Acad. Sci. U. S. A..

[cit29] Kang Y. S., Risbud S., Rabolt J. F., Stroeve P. (1996). Chem. Mater..

[cit30] Majewski P., Thierry B. (2007). Crit. Rev. Solid State Mater. Sci..

[cit31] Baumgartner J., Bertinetti L., Widdrat M., Hirt A. M., Faivre D. (2013). PLoS One.

[cit32] Nazrazadani S., Raman A. (1993). Corros. Sci..

[cit33] Kim W., Suh C.-Y., Cho S.-W., Roh K.-M., Kwon H., Song K., Shon I.-J. (2012). Talanta.

[cit34] Aghazadeh M., Karimzadeh I., Ganjali M. R. (2017). J. Magn. Magn. Mater..

[cit35] de Cogan F., Booth A., Gough J. E., Webb S. J. (2011). Angew. Chem., Int. Ed..

[cit36] Cheng F.-Y., Su C.-H., Yang Y.-S., Yeh C.-S., Tsai C.-Y., Wu C.-L., Wu M.-T., Shieh D.-B. (2005). Biomaterials.

[cit37] McGrath A. J., Dolan C., Cheong S., Herman D. A. J., Naysmith B., Zong F., Galvosas P., Farrand K. J., Hermans I. F., Brimble M., Williams D. E., Jin J., Tilley R. D. (2017). J. Magn. Magn. Mater..

[cit38] Jurašin D. D., Ćurlin M., Capjak I., Crnković T., Lovrić M., Babič M., Horák D., Vrček I. V., Gajović S. (2016). Beilstein J. Nanotechnol..

[cit39] Bhattacharjee S. (2016). J. Controlled Release.

[cit40] Bruvera I. J., Mendoza Zélis P., Pilar Calatayud M., Goya G. F., Sánchez F. H. (2015). J. Appl. Phys..

[cit41] Ozkaya T., Toprak M. S., Baykal A., Kavas H., Köseoğlu Y., Aktaş B. (2009). J. Alloys Compd..

[cit42] Barnakov Y. A., Yu M. H., Rosenzweig Z. (2005). Langmuir.

[cit43] Casula M. F., Corrias A., Arosio P., Lascialfari A., Sen T., Floris P., Bruce I. J. (2011). J. Colloid Interface Sci..

[cit44] Lee N., Hyeon T. (2012). Chem. Soc. Rev..

[cit45] Nagata Y., Burger M. M. (1974). J. Biol. Chem..

[cit46] Noble G. T., Flitsch S. L., Liem K. P., Webb S. J. (2009). Org. Biomol. Chem..

[cit47] Lee N., Kim H., Choi S. H., Park M., Kim D., Kim H.-C., Choi Y., Lin S., Kim B. H., Jung H. S., Kim H., Park K. S., Moon W. K., Hyeon T. (2011). Proc. Natl. Acad. Sci. U. S. A..

[cit48] Wang Y.-X. J. (2011). Quant. Imaging Med. Surg..

[cit49] Hu F., MacRenaris K. W., Waters E. A., Liang T., Schultz-Sikma E. A., Eckermann A. L., Meade T. J. (2009). J. Phys. Chem. C.

[cit50] Hachani R., Birchall M. A., Lowdell M. W., Kasparis G., Tung L. D., Manshian B. B., Soenen S. J., Gsell W., Himmelreich U., Gharagouzloo C. A., Sridhar S., Thanh N. T. K. (2017). Sci. Rep..

[cit51] Sittel I., Galan M. C. (2017). Org. Biomol. Chem..

[cit52] Gustafson H. H., Holt-Casper D., Grainger D. W., Ghandehari H. (2015). Nano Today.

[cit53] Kang J. H., Super M., Yung C. W., Cooper R. M., Domansky K., Graveline A. R., Mammoto T., Berthet J. B., Tobin H., Cartwright M. J., Watters A. L., Rottman M., Waterhouse A., Mammoto A., Gamini N., Rodas M. J., Kole A., Jiang A., Valentin T. M., Diaz A., Takahashi K., Ingber D. E. (2014). Nat. Med..

