# Supplementary material for: High-throughput chemical and chemoenzymatic approaches to saccharide-coated magnetic nanoparticles for MRI
Source: Nanoscale Adv. 2019 Jul 29;1(9):3597–606. doi: 10.1039/c9na00376b (PMC9417132; doi:10.1039/c9na00376b)
Supplement: NA-001-C9NA00376B-s001 [file NA-001-C9NA00376B-s001.pdf]

Electronic Supplementary Material (ESI) for Nanoscale Advances.
This journal is © The Royal Society of Chemistry 2019

Electronic supplementary information (ESI)

High-throughput chemical and chemoenzymatic approaches to saccharide-coated magnetic nanoparticles for MRI

Thomas W. Fallows,[1,2] Andrew J. McGrath,[3,4] Joana Silva,[1,2] Simon G. McAdams,[1,5] Andrea Marchesi,[1,2] Floriana Tuna,[1,6] Sabine L. Flitsch,[1,2] Richard D. Tilley,[3,4,7] Simon J. Webb.[1,2]*

1. *School of Chemistry, University of Manchester, Oxford Road, Manchester M13 9PL, United Kingdom.*
2. *Manchester Institute of Biotechnology, University of Manchester, 131 Princess St, Manchester M1 7DN, United Kingdom.*
3. *School of Chemistry, University of New South Wales, Australia.*
4. *Australian Centre for NanoMedicine, University of New South Wales, Australia*
5. *School of Materials, University of Manchester, Oxford Road, Manchester, United Kingdom.*
6. *Photon Science Institute, University of Manchester, Oxford Road, Manchester M13 9PL, United Kingdom.*
7. *Electron Microscope Unit, Mark Wainwright Analytical Centre, University of New South Wales, Australia.*

*. Email: S.Webb@manchester.ac.uk; Tel: +44 161 306 4524.

TABLE OF CONTENTS

1. General Methods and Equipment **4**

1.1 Abbreviations 4

2. Synthesis of Saccharide-Hydrazide Adducts **5**

2.1 General experimental procedure for adduct synthesis 5

2.2 Comparison of different basic catalysts 5

2.3 General procedure for adduct purification 6

2.4 General procedure for determining α:β ratio in adducts 7

2.5 Characterisation data for adducts **2** - **12** 9

2.5.1 3,4-Dihydroxy-*N*'-(1-deoxyglucopyranos-1-yl)-benzohydrazide **2** 9

2.5.2 3,4-Dihydroxy-*N*'-(4-*O*-(β-galactosyl)-1-deoxyglucopyranos-1-yl)-benzohydrazide **3** 9

2.5.3 3,4-Dihydroxy-*N*'-(1-deoxy-4-*O*-(3-*O*-(*N*-acetyl-α-neuraminosyl)-β-galactopyranosyl)glucopyranos-1-yl)-benzohydrazide **4** 9

2.5.4 3,4-Dihydroxy-*N*'-(1,2-dideoxy-2-(acetylamino)-glucopyranos-1-yl)-benzohydrazide **5** 10

2.5.5 3,4-Dihydroxy-*N*'-(1,2-dideoxyglucopyranos-1-yl)-benzohydrazide **6** 10

2.5.6 3,4-Dihydroxy-*N*'-(1-deoxymannopyranos-1-yl)-benzohydrazide **7** 10

2.5.7 3,4-Dihydroxy-*N*'-(1-deoxyfucopyranos-1-yl)-benzohydrazide **8** 11

2.5.8 3,4-Dihydroxy-*N*'-(4-*O*-(β-galactosyl)-1,2-dideoxy-2-(acetylamino)-glucopyranos-1-yl)-benzohydrazide **9** 12

2.5.9 3,4-Dihydroxy-*N*'-(1,2-dideoxy-2-(amino)-glucopyranos-1-yl)-benzohydrazide **10** 12

2.5.10 3,4-Dihydroxy-*N*'-(1-deoxygalactopyranos-1-yl)-benzohydrazide **11** 13

2.5.11 3,4-Dihydroxy-*N*'-(1-deoxy-6-deoxy-(6-phosphoryloxy)-glucopyranos-1-yl)-benzohydrazide disodium salt **12** 13

3. ^{1}H and ^{13}C NMR spectra of reported compounds 2 -5 **14**

3.1.1 ^{1}H NMR spectrum for glucose adduct **2** (11:89 α:β, CD_3OD) 14

3.1.2 ^{13}C NMR spectrum for glucose adduct **2** (11:89 α:β, CD_3OD) 14

3.1.3 ^{1}H NMR spectrum for lactose adduct **3** (10:90 α:β, CD_3OD) 15

3.1.4 ^{13}C NMR spectrum for lactose adduct **3** (10:90 α:β, CD_3OD) 15

3.1.5 ^{1}H NMR spectrum for sialyllactose adduct **4** (9:91 α:β, CD_3OD) 16

3.1.6 ^{13}C NMR spectrum for sialyllactose adduct **4** (9:91 α:β, CD_3OD) 16

3.1.7 ^{1}H NMR spectrum for *N*-acetylglucosamine adduct **5** (14:86 α:β, CD_3OD) 17

3.1.8 ^{13}C NMR spectrum for *N*-acetylglucosamine adduct **5** (14:86 α:β, CD_3OD) 17

4. ^{1}H and ^{13}C NMR spectra of new compounds 6 -12 **18**

4.1.1 ^{1}H NMR spectrum for 2-deoxyglucose adduct **6** (22:78 α:β, CD_3OD) 18

4.1.2 ^{13}C NMR spectrum for 2-deoxyglucose adduct **6** (22:78 α:β, CD_3OD) 18

4.1.3 ^{1}H NMR spectrum for mannose adduct **7** (>99 % β, CD_3OD) 19

4.1.4 ^{13}C NMR spectrum for mannose adduct **7** (>99 % β, CD_3OD) 19

4.1.5 ^{1}H NMR spectrum for L-fucose adduct **8** (19:81 α:β, CD_3OD) 20

4.1.6 ^{13}C NMR spectrum for L-fucose adduct **8** (19:81 α:β, CD_3OD) 20

4.1.7 ^{1}H NMR spectrum for *N*-acetyllactosamine adduct **9** (10:90 α:β, CD_3OD) 21

4.1.8 ^{13}C NMR spectrum for *N*-acetyllactosamine adduct **9** (10:90 α:β, CD_3OD) 21

4.1.9 ^{1}H NMR spectrum for glucosamine adduct **10** (>99% β, CD_3OD) 22

4.1.10 ^{13}C NMR spectrum for glucosamine adduct **10** (>99% β, CD_3OD) 22

4.1.11 ^{1}H NMR spectrum for galactose adduct **11** (15:85 α:β, CD_3OD) 23

4.1.12 ^{13}C NMR spectrum for galactose adduct **11** (15:85 α:β, CD_3OD) 23

4.1.13 ^{1}H NMR spectrum for glucose-6-phosphate adduct **12** (>99% β, D_2O) 24

4.1.14 ^{13}C NMR spectrum for glucose-6-phosphate adduct **12** (>99% β, D_2O) 24

5. XRD for uncoated MNPs and MNPs coated with 2-8 and 10-12. **25**

6. TEM measurements of uncoated MNP size distribution **26**

7. Inductively Coupled Plasma Atomic Emission Spectroscopy **27**

8. Estimation of the concentration of GlcNAc on 5-MNPs **27**

9. Additional MRI relaxation data **28**

9.1 Without WGA 28

9.2 With WGA 29

9.3 Plot of iron concentration against $1/T_1$ for GlcNAc-coated MNPs 29

10. Dynamic light scattering and zeta potential measurements **30**

11. References **34**

1. General Methods and Equipment

Reagents were purchased from Sigma-Aldrich Co. Ltd., Dorset, UK. In the case of 3,4-dihydroxybenzhydrazide **1**, the supplier was Fluorochem, Derbyshire. Permanent magnets were bought from e-magnets UK, Hertfordshire, UK.

Reversed-phase HPLC purification was performed on an Agilent 1100 series system with an Agilent Eclipse XDB-C18 (9.4 mm × 250 mm) column. Sonication of nanoparticles during the coating process was performed with a Sonics VCX130PB Ultrasonic Processor with a stepped micro tip (3 mm × 136 mm) at 20 kHz.

NMR spectra were recorded in deuterated solvents using a Brüker 400 MHz Avance spectrometer with broadband probe or a Brüker 800 MHz Avance III. NMR chemical shift values are referenced to residual peaks from non-deuterated solvent and measured in ppm. Splitting patterns are reported as singlets (s), doublets (d), triplets (t), quartets (q), multiplets (m) or a combination of the above and coupling constants are measured in Hz. Electrospray mass spectrometry was performed on a Micromass LCT instrument using a Waters 2790 separations module with electrospray ionization and TOF fragment detection. High resolution mass spectrometry was performed on a Water Q-TOF micro with an ES+/- ion source. Elemental analysis was performed using a Thermo Scientific FLASH 2000 series CHNS/O Analyser. DLS and zeta potential measurements were carried out using a Malvern Zetasizer Nano.

1.1 Abbreviations

FC, field cooled; HPLC, high performance liquid chromatography; ICP-AES, inductively coupled plasma atomic emission spectroscopy; MNP, magnetic nanoparticle; NMR, nuclear magnetic resonance; SQUID, superconducting quantum interference device; TEM, transmission electron microscopy; THF, tetrahydrofuran; XRD, X-ray diffraction; ZFC, zero field cooled.

2. Synthesis of Saccharide-Hydrazide Adducts

2.1 General experimental procedure for adduct synthesis

The synthesis of compounds **2**-**12** was performed according to the following general procedure.

The saccharide (0.3 mmol) and 3,4-dihydroxybenzhydrazide **1** (0.3 mmol) were added to methanol (10 mL) containing aniline (5 mM from stock solution) and heated to reflux at 65 °C under nitrogen with stirring overnight. The solvent was removed *in vacuo* and the crude product was dissolved in a minimum of water, filtered if necessary to remove particulate material and purified by HPLC (see Section 2.3). The product containing fractions were collected and freeze dried to give the product as a white powder in all cases.

2.2 Comparison of different basic catalysts

NMR tube reactions were carried out in d_4-methanol in order to assess the effectiveness of each catalyst. Each tube was filled with 3,4-dihydroxybenzhydrazide **1** (3 mg, 0.018 mmol) and glucose (4.8 mg, 0.018 mmol), as well a 5 mM stock solution of the catalyst in d_4-methanol (0.6 mL). A control was also set up containing **1**, glucose and d_4-methanol with no catalyst present. ^{1}H NMR spectra were measured at 0 hours and the NMR tubes were then placed in an oil bath at 65 °C. Further ^{1}H NMR spectra were measured at 1, 2, 4, 6, 8 and 24 h. The yields were calculated by integrating the anomeric peaks of glucose and the glucose adduct **2**. Taking the integrals of the α- and β-anomers of the product and dividing by the integrals of all glucose anomeric peaks (i.e. α and β for both product and starting material) gave a crude relative yield which could be converted into a percentage yield by multiplying by 100.

The aniline-catalysed reaction produced the product in better yields that the uncatalysed reaction. Both *p*-phenylenediamine and anthranilic acid catalysed the reaction much better than aniline (Figure S2.1) with yields in excess of 80%, comparable to values previously described in the literature.[1,2]

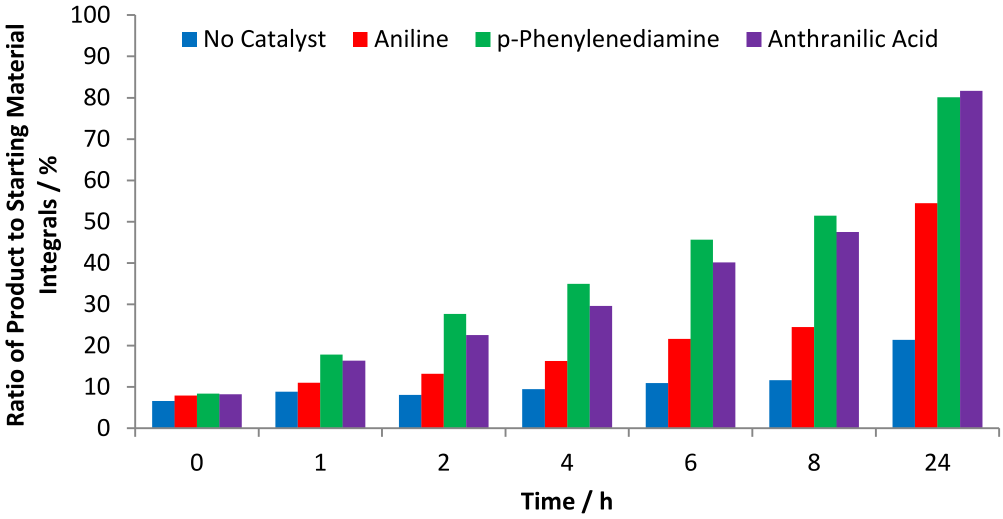

Figure S2.1: Chart showing the change in integral ratio for the sum of α and β anomeric resonances for **2** compared to **2** + glucose in the ^{1}H NMR spectra of glucose mixed with 3,4-dihydroxybenzhydrazide **1** in fresh solvent with different catalysts. Each solution was heated at 65 °C over 24 h. Ratio calculated [anomeric peaks of **2** / (anomeric peaks of **2** + anomeric peaks of glucose) × 100].

2.3 General procedure for adduct purification

^{1}H NMR spectra of the crude products (e.g. the glucose adduct **2**, Figure S2.2) showed that the reaction did not produce many by-products, although the reaction does not go to completion after 24 h. ^{1}H NMR spectroscopy shows the crude product as a mixture of anomers as well as the anomeric peaks from the starting saccharide and the aromatic peaks from the starting hydrazide **1**. The peaks from the catalyst can also be observed further downfield (Figure S2.2 inset), although they are sometimes obscured by the aromatic peaks of the benzhydrazide **1**.

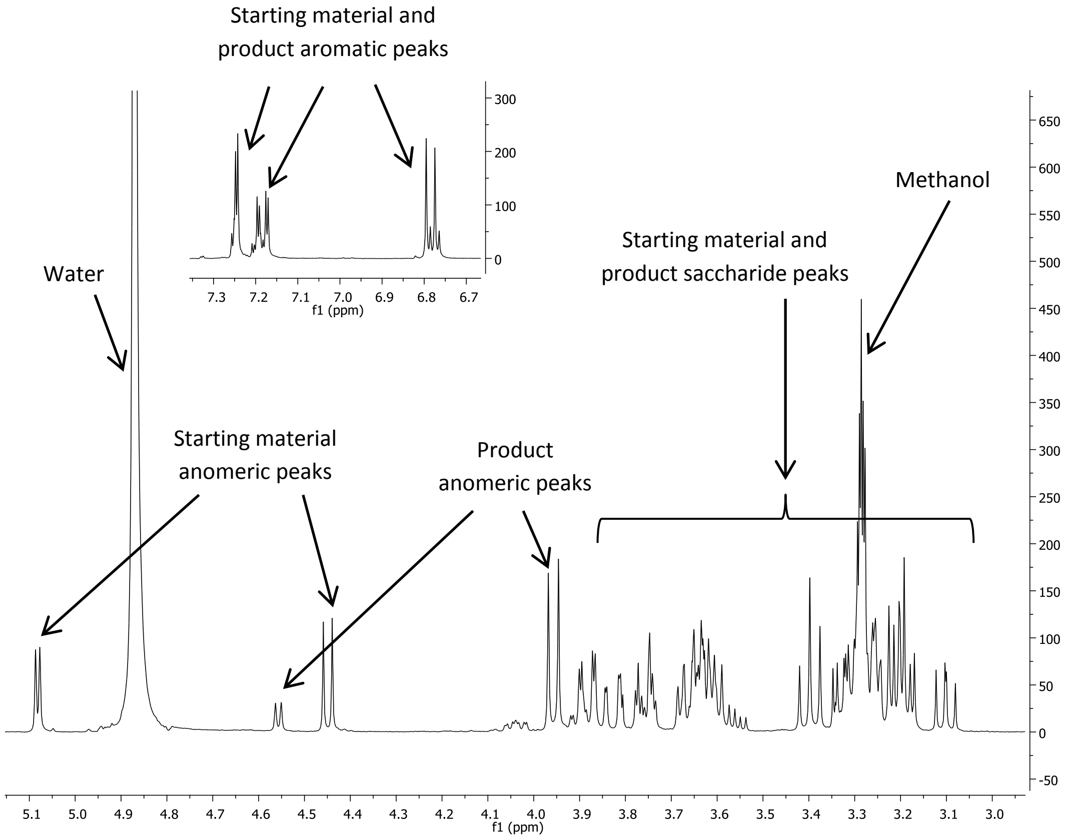

Figure S2.2: Labelled crude ^{1}H NMR spectrum of glucose-hydrazide adduct **2** in d_4-methanol from 2.95 to 5.15 ppm. Inset: Region from 6.65 to 7.35 ppm.

It was possible to remove some unreacted hydrazide **1** from the crude reaction mixture by first removing the methanol under reduced pressure before redissolving the crude reaction mixture in Milli-Q filtered water (5 mL), causing some of the hydrazide to precipitate out, and filtering through cotton wool. However, this did not remove all of the hydrazide and did not remove unreacted saccharide and catalyst from the filtrate. For this reason the reaction mixture (typically ~60 mg of crude material) was purified by high-performance liquid chromatography (HPLC) in the reversed-phase mode. The non-polar stationary phase was a C_{18} column and the polar mobile phase was a mixture of water and tetrahydrofuran (THF) (v/v 95%/5%). A semi-preparative column (9.4 mm × 250 mm) was used and through purification of several aliquots of 0.5 mL, the entire crude reaction mixture of approximately 60 mg could usually be purified during one day. In order to elute less polar compounds, the proportion of THF (the less polar solvent) was increased from 5% to 50% over 40 mins. Typical retention times for monosaccharide adducts were from 14 to 18 mins at 1 mL/min. The column was then washed with a mixture of water/THF 5%/95% for a further 30 mins to remove all remaining material before the next crude sample was purified.

The collected fractions were then concentrated under reduced pressure in order to remove the THF, before being lyophilised to give the products, as a mixture of α and β anomers, as white powders. In all, eleven small saccharides were used to form the library of saccharide adducts **2**-**12** (Figure S2.3).

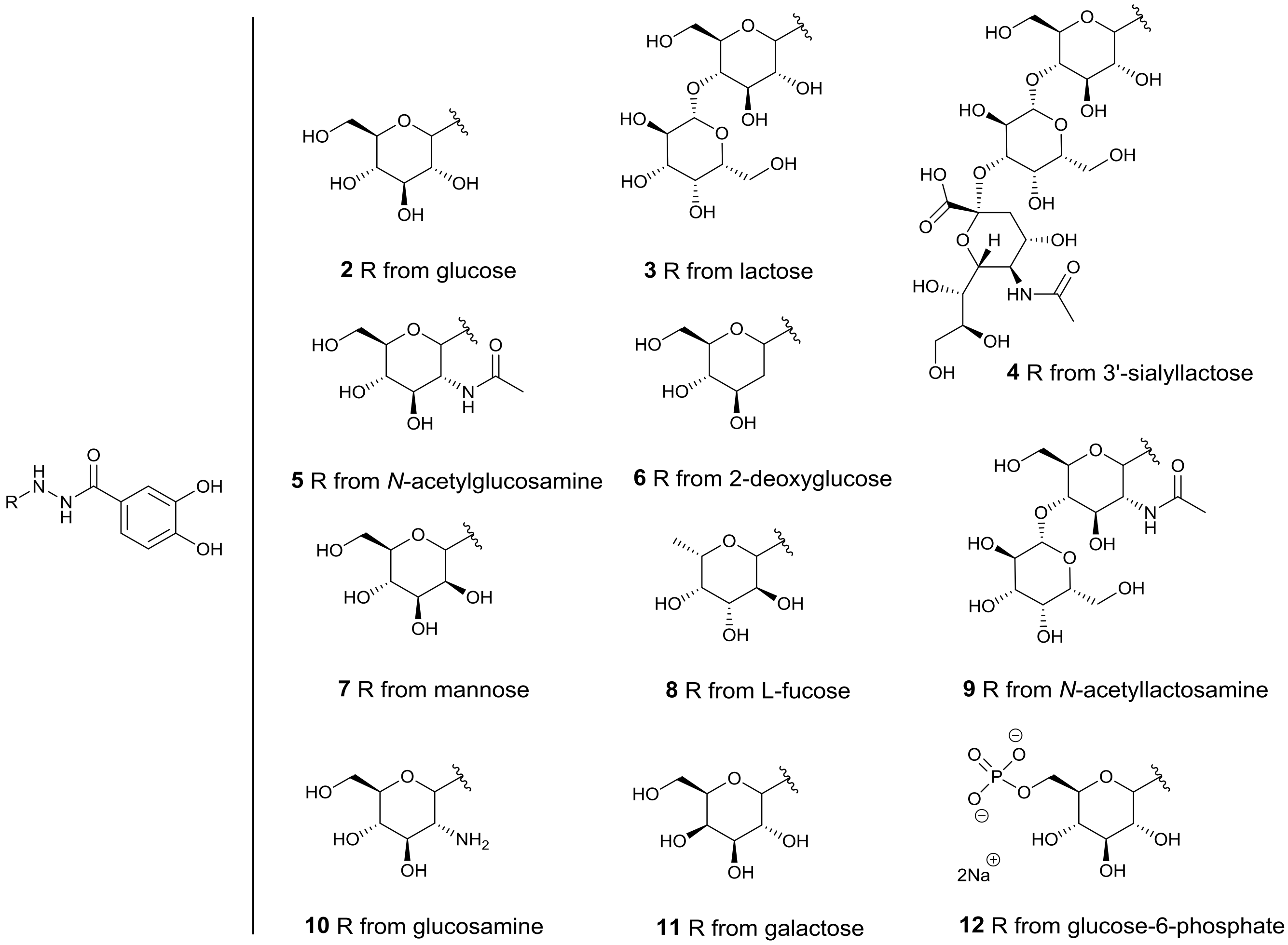

Figure S2.3: Structures of saccharide hydrazide adducts **2**-**12**. Adducts **2** – **5** reported previously.[3]

2.4 General procedure for determining α:β ratio in adducts

The α:β anomeric ratios in saccharide adducts were determined using ^{1}H NMR spectroscopy. The peaks corresponding to the α and β anomer signals were integrated and a percentage for each was calculated using either (α/(α + β)) × 100 or (β/(α + β)) × 100.

For example, in the case of **2** the α anomer was calculated to be (0.11/(0.11 + 0.92)) × 100 = 11% and the β anomer was calculated to be (0.92/(0.11 + 0.92)) × 100 = 89% (Figure S2.4).

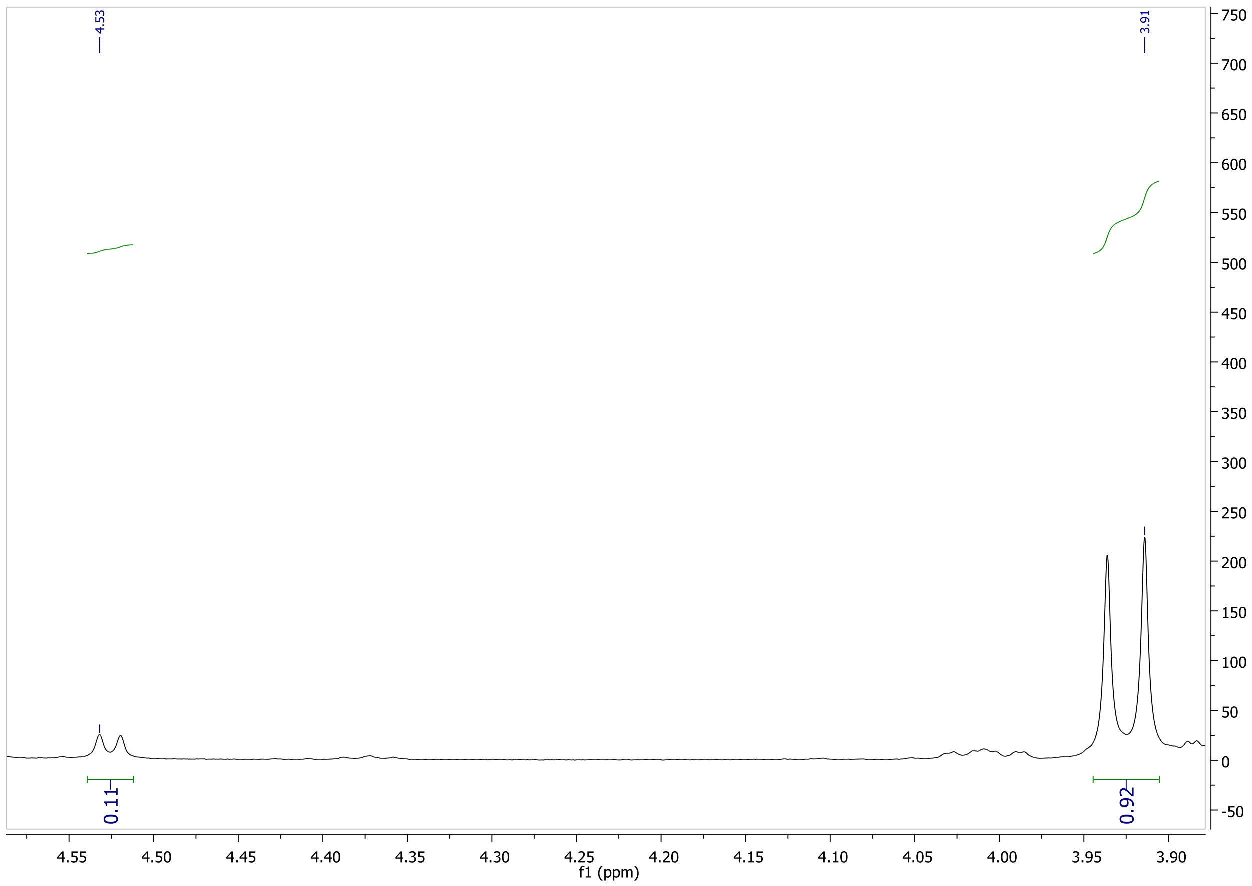

Figure S2.4: ^{1}H NMR spectrum of saccharide adduct **2** showing the integrals of the anomeric peaks, with the α anomer at 4.53 ppm and the β anomer at 3.93 ppm.

2.5 Characterisation data for adducts 2 - 12

In each case the reported NMR spectroscopy data is for both anomers, but usually only the major β anomer is detected in the ^{13}C NMR spectra.

2.5.1 3,4-Dihydroxy-*N*'-(1-deoxyglucopyranos-1-yl)-benzohydrazide 2

Yield: 79 mg, 75%, 11:89 mixture of α/β-anomers in methanol-d_4. Spectroscopic data matched that previously reported.[3]

^{1}H NMR (400 MHz, CD_3OD): *δ* 7.21 (d, 1H, *J* = 2.1 Hz, **7**), 7.15 (dd, 1H, J_1 = 8.3 Hz, J_2 = 2.2 Hz, **8**), 6.75 (d, 1H, *J* = 8.3 Hz, **9**), 4.53 (d, 0.11H, *J* = 4.9 Hz, **1 α**), 3.92 (d, 0.89H, *J* = 8.8 Hz, **1 β**), 3.85 (dd, 1H, J_1 = 11.7 Hz, J_2 =2.3 Hz, **6a**), 3.58 (dd, 1H, J_1 = 11.6 Hz, J_2 = 6.5 Hz, **6b**), 3.37 (t, 1H, *J* = 9.0 Hz, **3**), 3.29-3.23 (m, MeOH obscuring **5**), 3.20-3.13 (m, 2H, **2**, **4**).

^{13}C NMR (101 MHz, CD_3OD): *δ* 170.4, 150.5, 146.4, 125.2, 120.9, 115.9 (2C), 92.4, 79.0, 78.3, 72.6, 71.7, 63.0.

HRMS for $[(C_{13}H_{18}N_2O_8) + H^+]^+$ expected 331.1136 found 331.1137.

Anal. Calcd. For $C_{13}H_{18}N_2O_8$ (+ H_2O): C, 44.83, H, 5.79, N, 8.04. Found: C, 44.71, H, 6.19, N, 7.82.

2.5.2 3,4-Dihydroxy-*N*'-(4-*O*-(β-galactosyl)-1-deoxyglucopyranos-1-yl)-benzohydrazide 3

Yield: 109 mg, 74%, 10:90 mixture of α/β-anomers in methanol-d_4. Spectroscopic data matched that previously reported.[3]

^{1}H NMR (400 MHz, CD_3OD): *δ* 7.24 (d, 1H, *J* = 2.0 Hz, **13**), 7.17 (dd, 1H, J_1 = 8.3 Hz, J_2 = 2.0 Hz, **14**), 6.78 (d, 1H, J_1 = 8.3 Hz, **15**), 4.57 (d, 0.10H, *J* = 5.1 Hz, **1 α**), 4.29 (d, 1H, *J* = 7.6 Hz, **7**), 4.00 (d, 0.90H, *J* = 8.9 Hz, **1 β**), 3.92 (dd, 1H, J_1 = 11.8 Hz, J_2 = 1.8 Hz, **6a**), 3.81-3.71 (m, 3H, **5**, **12a**, **12b**), 3.65 (dd, 1H, J_1 = 11.7 Hz, J_2 = 4.5 Hz, **6b**), 3.59-3.40 (m, 6H, **3**, **4**, **8**, **9**, **10**, **11**), 3.29-3.24 (MeOH peak obscuring **2**).

^{13}C NMR (101 MHz, CD_3OD): *δ* 170.5, 150.6, 146.4, 125.2, 120.9, 116.4, 115.9, 105.1, 92.2, 80.4, 77.7, 77.1, 76.6, 74.8, 72.5, 72.2, 70.3, 62.5, 62.1.

HRMS for $[(C_{19}H_{28}N_2O_{13})+ H^+]^+$ expected 493.1664 found 493.1666.

2.5.3 3,4-Dihydroxy-*N*'-(1-deoxy-4-*O*-(3-*O*-(*N*-acetyl-α-neuraminosyl)-β-galactopyranosyl)glucopyranos-1-yl)-benzohydrazide 4

The synthesis of glycoadduct **4** was performed on a smaller scale, using 0.12 mmol of hydrazide and saccharide.

Yield: 61 mg, 63%, 9:91 mixture of α/β-anomers in methanol-d_4. Spectroscopic data matched that previously reported.[3]

^{1}H NMR (400 MHz, CD_3OD): *δ* 7.25 (d, 1H, *J* = 2.1 Hz, **21**), 7.19 (dd, 1H, J_1 = 8.3 Hz, J_2 = 2.1 Hz, **22**), 6.78 (d, 1H, *J* = 8.3 Hz, **23**), 4.47 (d, 0.09H, *J* = 7.8 Hz, **1 α**), 4.36 (d, 1H, *J* = 7.8 Hz, **7**), 4.05-3.98 (m, 1.91H, **1 β**, **16**), 3.95 (dd, 1H, J_1 = 11.7 Hz, J_2 = 2.1 Hz, **6a**), 3.90-3.40 (m, 18H, **2**-**5**, **6b**, **8**-**12**, **14**-**15**, **17**-**19**), 2.83 (ddd, 1H, J_1 = 12.4 Hz, J_2 = 2.1 Hz, J_3 = 2.1 Hz, **13a**), 1.98 (s, 3H, **20**), 1.70 (ddd, 1H, J_1 = 11.0 Hz, J_2 = 8.0 Hz, J_3 = 3.2 Hz, **13b**).

^{13}C NMR (101 MHz, CD_3OD): δ 175.5, 174.9, 170.7, 150.5, 146.4, 125.2, 121.0, 116.0, 115.9, 105.1, 101.0, 91.8, 80.6, 77.7, 77.6, 77.1, 76.5, 74.9, 73.0, 72.0, 70.7, 70.1, 69.3, 69.0, 64.6, 62.7, 61.9, 53.9, 42.1, 22.6.

HRMS for $[(C_{30}H_{45}N_3O_{21}) + H^+]^+$ expected 784.2618 found 784.2604.

2.5.4 3,4-Dihydroxy-*N*'-(1,2-dideoxy-2-(acetylamino)-glucopyranos-1-yl)-benzohydrazide 5

Yield: 82 mg, 70%, 14:86 mixture of α/β-anomers in methanol-d_4. Spectroscopic data matched that previously reported.[3]

^{1}H NMR (400 MHz, CD_3OD): δ 7.26 (d, 1H, J = 2.1 Hz, **8**), 7.19 (dd, 1H, J_1 = 8.3 Hz, J_2 = 2.1 Hz, **9**), 6.79 (d, 1H, J = 8.3 Hz, **10**), 4.60 (d, 0.14H, J = 4.8 Hz, **1 α**), 4.02 (d, 0.86H, J = 9.5 Hz, **1 β**), 3.83 (dd, 1H, J_1 = 12.0 Hz, J_2 = 1.9 Hz, **6a**), 3.76 (t, 1H, J = 9.9 Hz, **2**), 3.58 (dd, 1H, J_1 = 12.0 Hz, J_2 = 6.3 Hz, **6b**), 3.46 (t, 1H, J = 9.0 Hz, **3**), 3.26-3.17 (m, 2H, **5**, **4**), 2.05 (s, 3H, **7**).

^{13}C NMR (101 MHz, CD_3OD): δ 175.2, 168.6, 150.5, 146.5, 125.2, 120.5, 116.0, 115.5, 91.4, 79.3, 76.3, 72.4, 63.1, 54.7, 22.9.

HRMS for $[(C_{15}H_{21}N_3O_8) + Na^+]^+$ expected 394.1221 found 394.1217.

2.5.5 3,4-Dihydroxy-*N*'-(1,2-dideoxyglucopyranos-1-yl)-benzohydrazide 6

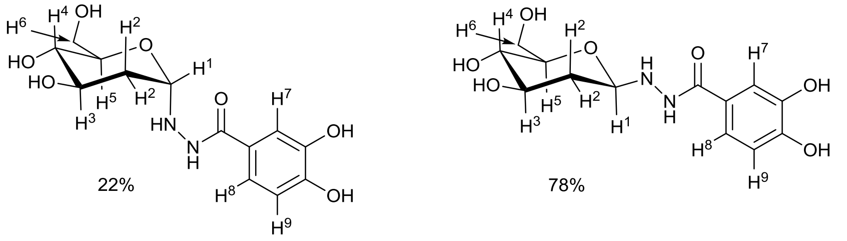

Yield: 30 mg, 61%, 22:78 mixture of α/β-anomers in methanol-d_4.

^{1}H NMR (400 MHz, CD_3OD): δ 7.23 (d, 1H, J = 2.1 Hz, **7**), 7.16 (dd, 1H, J_1 = 8.3 Hz, J_2 = 2.0 Hz, **8**), 6.77 (d, 1H, J = 8.3 Hz, **9**), 4.68 (d, 0.22H, J_1 = 4.5, **1 α**), 4.23 (dd, 0.78H, J_1 = 10.8, J_2 = 1.7 Hz, **1 β**), 3.87 (dd, 1H, J_1 = 11.5 Hz, J_2 = 2.3 Hz, **6a**), 3.63 (dd, 1H, J_1 = 11.6 Hz, J_2 = 6.3 Hz, **6b**), 3.60-3.53 (m, 1H, **3**), 3.21 (ddd, 1H, J_1 = 9.5 Hz, J_2 = 6.3 Hz, J_3 = 2.4 Hz, **5**), 3.11 (t, 1H, J = 9.5 Hz, **4**), 2.18 (ddd, 1H, J_1 = 12.5 Hz, J_2 = 5.1 Hz, J_3 = 1.5 Hz, **2a**), 1.46 (dt, 1H, J_1 = 12.4 Hz, J_2 = 11.4 Hz, **2b**).

^{13}C NMR (101 MHz, CD_3OD): δ 169.9, 150.2, 146.4, 120.8, 120.4, 115.9, 115.6, 88.6, 79.0, 73.2, 73.1, 63.2, 37.8.

HRMS for $[(C_{13}H_{18}N_2O_7) + Na^+]^+$ expected 337.1006 found 337.1002.

2.5.6 3,4-Dihydroxy-*N*'-(1-deoxymannopyranos-1-yl)-benzohydrazide 7

The synthesis of glycoadduct **7** was performed on a smaller scale, using 0.24 mmol of hydrazide and saccharide.

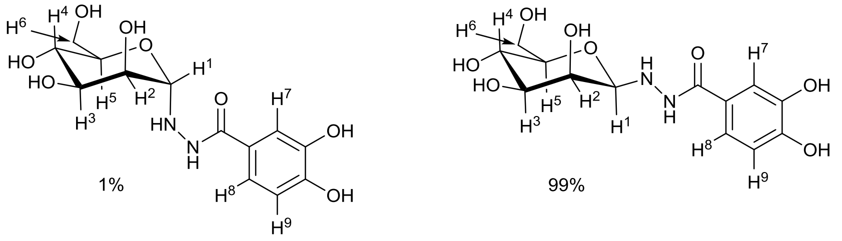

Yield: 45 mg, 57%, >99 % β-anomer in methanol-d_4.

^{1}H NMR (400 MHz, CD_3OD): δ 7.23 (d, 1H, J = 2.1 Hz, **7**), 7.17 (dd, 1H, J_1 = 8.3 Hz, J_2 = 2.2 Hz, **8**), 6.78 (d, 1H, J = 8.3 Hz, **9**), 4.17 (d, 1H, J = 0.7 Hz, **1 β**), 3.99 (d, 1H, J = 2.8 Hz, **2**), 3.89 (dd, 1H, J_1 = 11.6 Hz, J_2 = 2.4 Hz, **6a**), 3.63 (dd, 1H, J_1 = 11.6 Hz, J_2 = 7.2 Hz, **6b**), 3.51-3.42 (m, 2H, **3**, **4**), 3.20 (ddd, 1H, J_1 = 8.7 Hz, J_2 = 7.0 Hz, J_3 = 2.3 Hz, **5**).

^{13}C NMR (101 MHz, D_2O + CD_3OD): δ 170.1, 149.5, 145.3, 125.3, 121.5, 116.5, 115.9, 89.0, 78.5, 74.8, 71.0, 68.3, 62.4.

HRMS for $[(C_{13}H_{18}N_2O_8) + H^+]^+$ expected 331.1136 found 331.1148.

2.5.7 3,4-Dihydroxy-*N*'-(1-deoxyfucopyranos-1-yl)-benzohydrazide 8

The synthesis of glycoadduct **8** was performed on a smaller scale, using 0.16 mmol of hydrazide and saccharide.

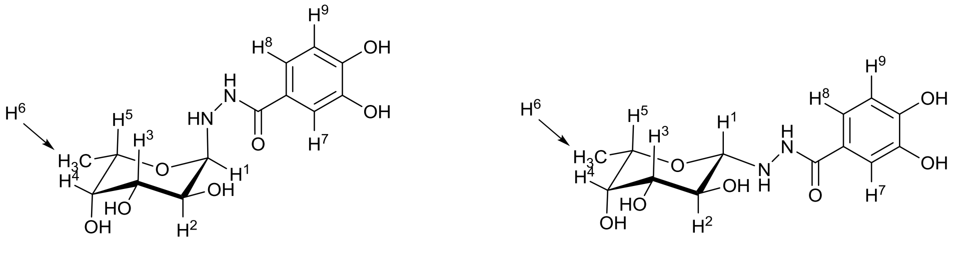

19% 81%

Yield: 24 mg, 45%, 19:81 mixture of α/β-anomers in methanol-d_4.

^{1}H NMR (400 MHz, CD_3OD): δ 7.23 (d, 1H, J = 2.2 Hz, **7**), 7.17 (dd, 1H, J_1 = 8.2 Hz, J_2 = 2.2 Hz, **8**), 6.77 (d, 1H, J = 8.3 Hz, **9**), 4.58 (d, 0.19H, J = 5.0 Hz, **1 α**), 3.91 (d, 0.81H, J = 8.3 Hz, **1 β**), 3.63 (qd, 1H, J_1 = 6.5 Hz, J_2 = 0.9 Hz, **5**), 3.58 (dd, 1H, J_1 = 3.1 Hz, J_2 = 0.9 Hz, **4**), 3.53-3.45 (m, 2H, **3**, **2**), 1.24 (d, 3H, J = 6.4 Hz, **6**).

^{13}C NMR (101 MHz, CD_3OD): δ 170.5, 150.6, 146.4, 125.3, 120.9, 115.9, 115.9, 92.7, 75.4, 73.4, 73.2, 69.5, 17.0.

HRMS for $[(C_{13}H_{18}N_2O_7) + Na^+)^+$ expected 337.1006 found 337.1025.

2.5.8 3,4-Dihydroxy-*N*'-(4-*O*-(β-galactosyl)-1,2-dideoxy-2-(acetylamino)-glucopyranos-1-yl)-benzohydrazide 9

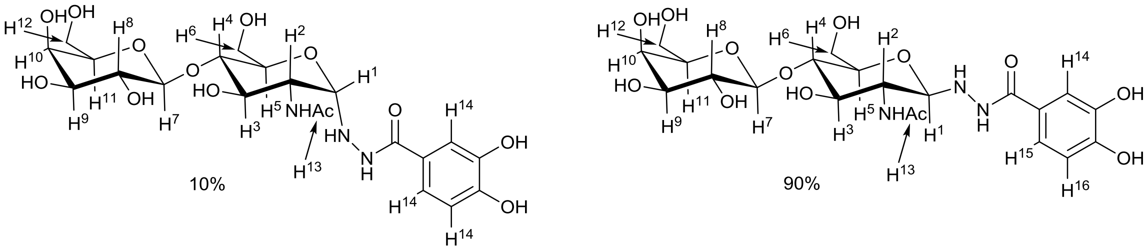

The synthesis of glycoadduct **9** was performed on a smaller scale, using 0.07 mmol of hydrazide and saccharide.

Yield: 3.1 mg, 8%, 10:90 mixture of α/β-anomers in methanol-d_4.

^{1}H NMR (400 MHz, CD_3OD): δ 7.26 (d, 1H, J = 2.1 Hz, **14**), 7.19 (dd, 1H, J_1 = 8.3 Hz, J_2 = 2.1 Hz, **15**), 6.78 (d, 1H, J = 8.3 Hz, **16**), 4.63 (d, 0.10H, J = 4.5 Hz, **1 α**), 4.29 (d, 1H, J = 7.5 Hz, **7**), 4.04 (d, 0.90H, J = 9.5 Hz, **1 β**), 3.89 (dd, 1H, J_1 = 12.0 Hz, J_2 = 2.1 Hz, **6a**), 3.84 (t, 1H, J = 9.7 Hz, **2**), 3.78-3.69 (m, 3H, **6b**, **12**), 3.67-3.60 (m, 2H, **3**, **10**), 3.56-3.39 (m, 5H, **4**, **5**, **8**, **9**, **11**), 2.03 (s, 3H, **13**).

^{13}C NMR (101 MHz, CD_3OD): δ 175.0, 173.6, 150.5, 146.5, 125.2, 120.5, 116.0, 115.5, 111.2, 105.1, 81.1, 78.0, 77.1, 76.4, 74.8, 74.4, 72.6, 70.3, 62.5, 54.1, 22.9.

HRMS for $[(C_{21}H_{31}N_3O_{13}) + Na^+]^+$ expected 556.1749 found 556.1760.

2.5.9 3,4-Dihydroxy-*N*'-(1,2-dideoxy-2-(amino)-glucopyranos-1-yl)-benzohydrazide 10

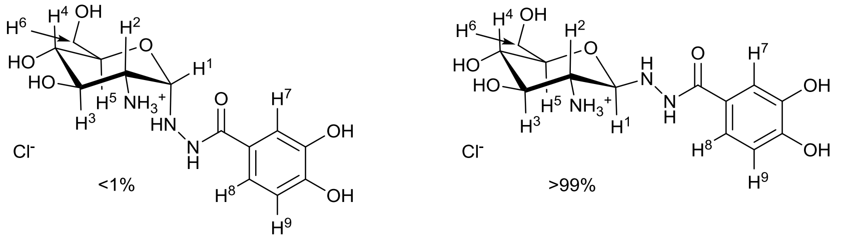

Yield: 30 mg, 30%, >99% β-anomer in methanol-d_4.

^{1}H NMR (400 MHz, CD_3OD): δ 7.26 (d, 1H, J = 2.2 Hz, **7**), 7.21 (dd, 1H, J_1 = 8.3 Hz, J_2 = 2.2 Hz, **8**), 6.80 (d, 1H, J = 8.3 Hz, **9**), 4.26 (d, 1H, J = 9.6 Hz, **1 β**), 3.90 (dd, 1H, J_1 = 11.6 Hz, J_2 = 2.3 Hz, **6a**), 3.67 (dd, 1H, J_1 = 11.6 Hz, J_2 = 6.0 Hz, **6b**), 3.52 (dd, 1H, J_1 = 10.0 Hz, J_2 = 8.6 Hz, **3**), 3.35 (ddd, 1H, J_1 = 9.7 Hz, J_2 = 6.0 Hz, J_3 = 2.3 Hz, **5**), 3.25 (dd, 1H, J_1 = 9.6 Hz, J_2 = 8.7 Hz, **4**), 2.82 (t, 1H, J = 10.0 Hz, **2**).

^{13}C NMR (101 MHz, CD_3OD): δ 181.9, 150.9, 146.5, 124.7, 121.2, 116.1, 115.9, 88.2, 79.5, 74.7, 71.8, 62.5, 54.7.

HRMS for $[C_{13}H_{20}N_3O_7]^+$ expected 330.1296 found 330.1291.

Anal. Calcd. For $C_{13}H_{19}N_3O_7$ (+ HCl, +2 H_2O): C, 38.86, H, 6.02, N, 10.46, Cl, 8.82. Found: C, 38.65, H, 5.83, N, 10.26, Cl, 9.35.

2.5.10 3,4-Dihydroxy-*N*'-(1-deoxygalactopyranos-1-yl)-benzohydrazide 11

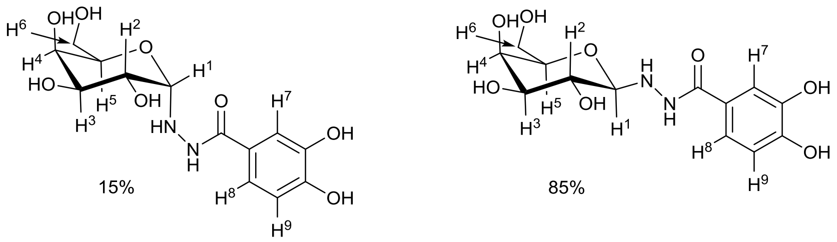

Yield: 46 mg, 45%, 15:85 mixture of α/β-anomers in methanol-d_4.

^{1}H NMR (400 MHz, CD_3OD): δ 7.28 (d, 1H, J = 1.4 Hz, **7**), 7.22 (dd, 1H, J_1 = 8.3 Hz, J_2 = 1.4 Hz, **8**), 6.81 (d, 1H, J = 8.2 Hz, **9**), 4.65 (d, 0.15H, J = 5.6 Hz, **1 α**), 3.94 (d, 0.85H, J = 8.6 Hz, **1 β**), 3.85-3.79 (m, 2H, **4**, **6a**), 3.58 (dd, 1H, J_1 = 11.3 Hz, J_2 = 4.1 Hz, **6b**), 3.60-3.53 (m, 3H, **2**, **3**, **5**).

^{13}C NMR (101 MHz, CD_3OD): δ 170.4, 150.5, 146.4, 125.4, 120.9, 115.9, 115.9, 93.2, 78.0, 75.3, 70.6, 70.0, 63.1.

HRMS for $[(C_{13}H_{18}N_2O_8) + H^+]^+$ expected 331.1136 found 331.1132.

2.5.11 3,4-Dihydroxy-*N*'-(1-deoxy-6-deoxy-(6-phosphoryloxy)-glucopyranos-1-yl)-benzohydrazide disodium salt 12

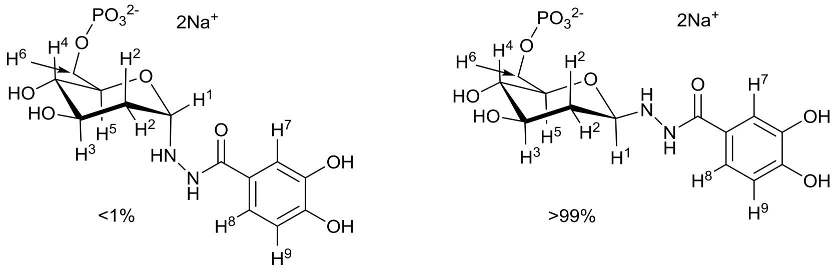

Yield: 73 mg, 56%, >99% β-anomer in deuterium oxide.

^{1}H NMR (400 MHz, D_2O): δ 7.20 (d, 1H, J = 1.8 Hz, **7**), 7.16 (dd, 1H, J_1 = 8.3 Hz, J_2 = 1.8 Hz, **8**), 6.83 (d, 1H, J_1 = 8.3 Hz, **9**), 4.14 (d, 1H, J = 9.0 Hz, **1 β**), 4.00-3.95 (m, 2H, **6a**, **6b**), 3.54-3.49 (m, 2H, **3**, **4**), 3.47-3.42 (m, 1H, **5**), 3.33 (dd, 1H, J_1 = 8.8 Hz, J_2 = 4.4 Hz, **2**).

^{13}C NMR (101 MHz, D_2O): δ 170.2, 151.1, 145.1, 122.2, 120.7, 116.0, 114.5, 90.0, 76.5, 75.7, 70.8, 69.1, 62.9.

HRMS for $[(C_{13}H_{17}N_2O_{11}P)^{2-} + H^+ + 2Na^+)^+$ expected 455.0438 found 455.0433.

Anal. Calcd. For $C_{13}H_{17}N_2O_{11}PNa_2$ (+ $3H_2O$): C, 30.72, H, 4.56, N, 5.51. Found: C, 30.60, H, 4.17, N, 5.21.

3. ^{1}H and ^{13}C NMR spectra of reported compounds 2 -5

3.1.1 ^{1}H NMR spectrum for glucose adduct 2 (11:89 α:β, CD_3OD)

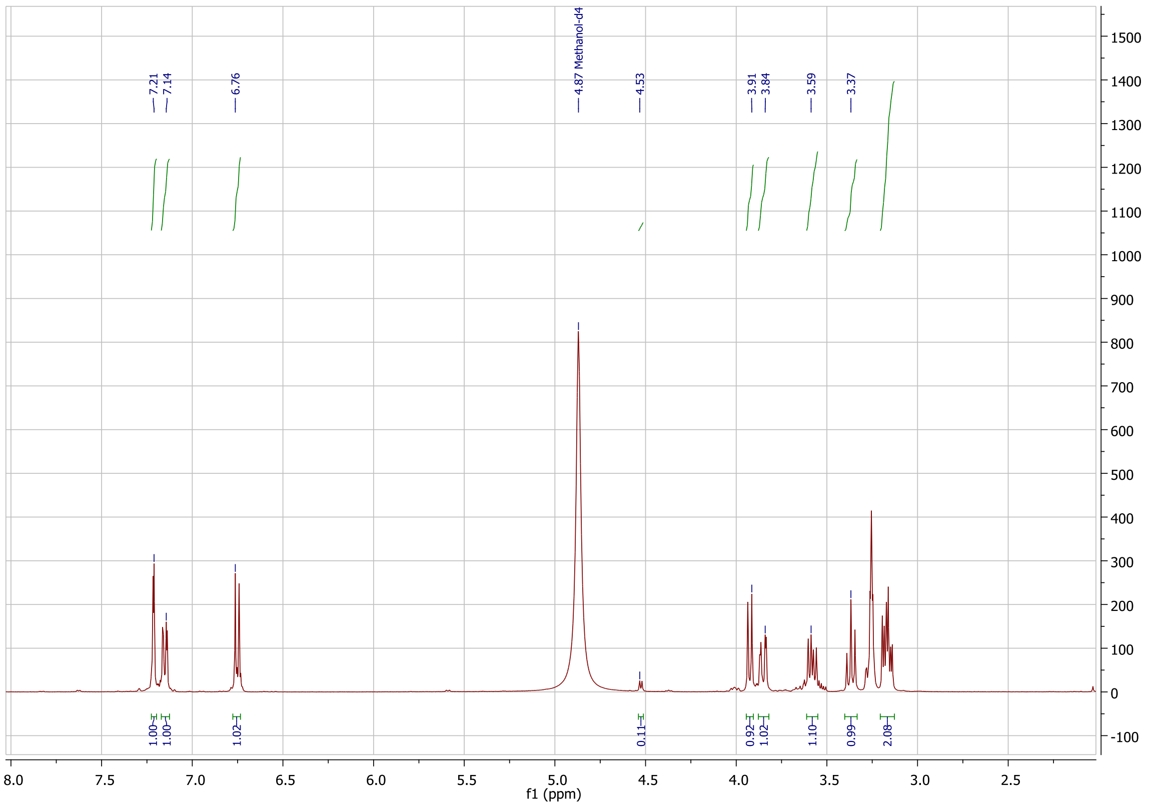

3.1.2 ^{13}C NMR spectrum for glucose adduct 2 (11:89 α:β, CD_3OD)

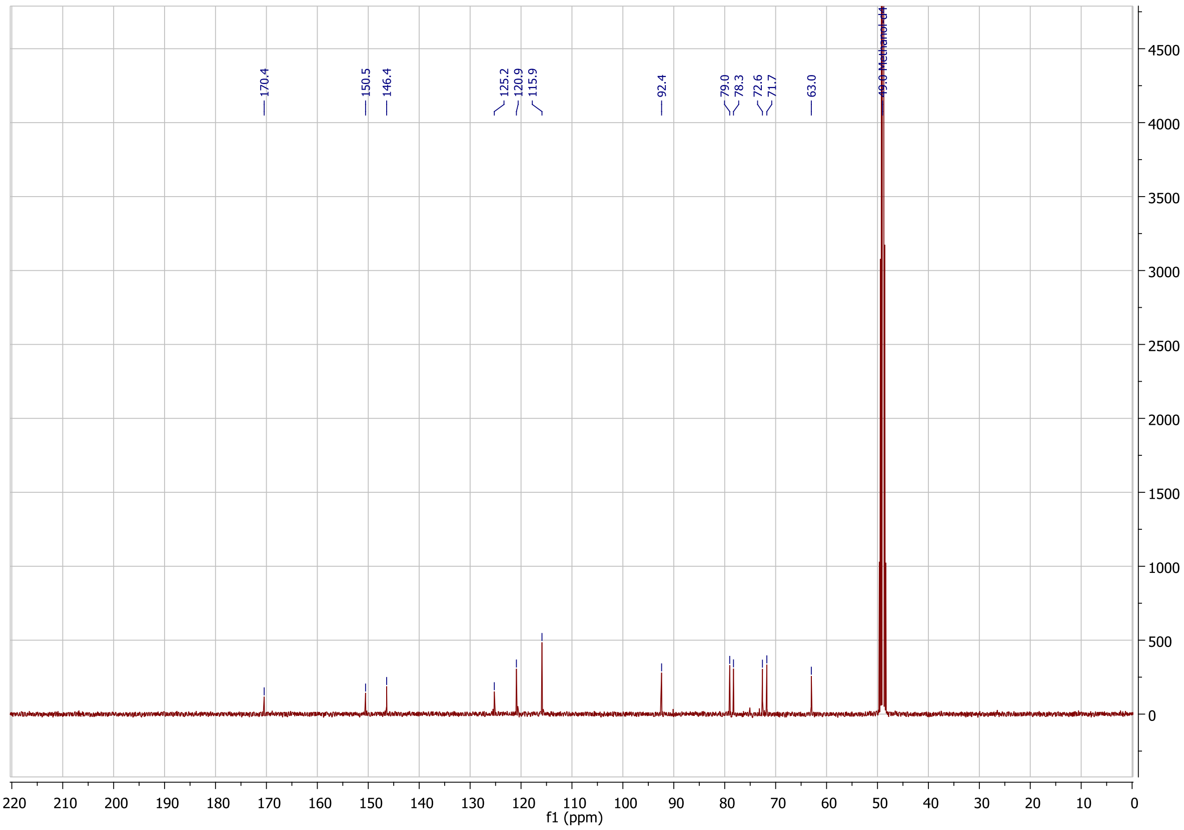

3.1.3 ^{1}H NMR spectrum for lactose adduct 3 (10:90 α:β, CD_3OD)

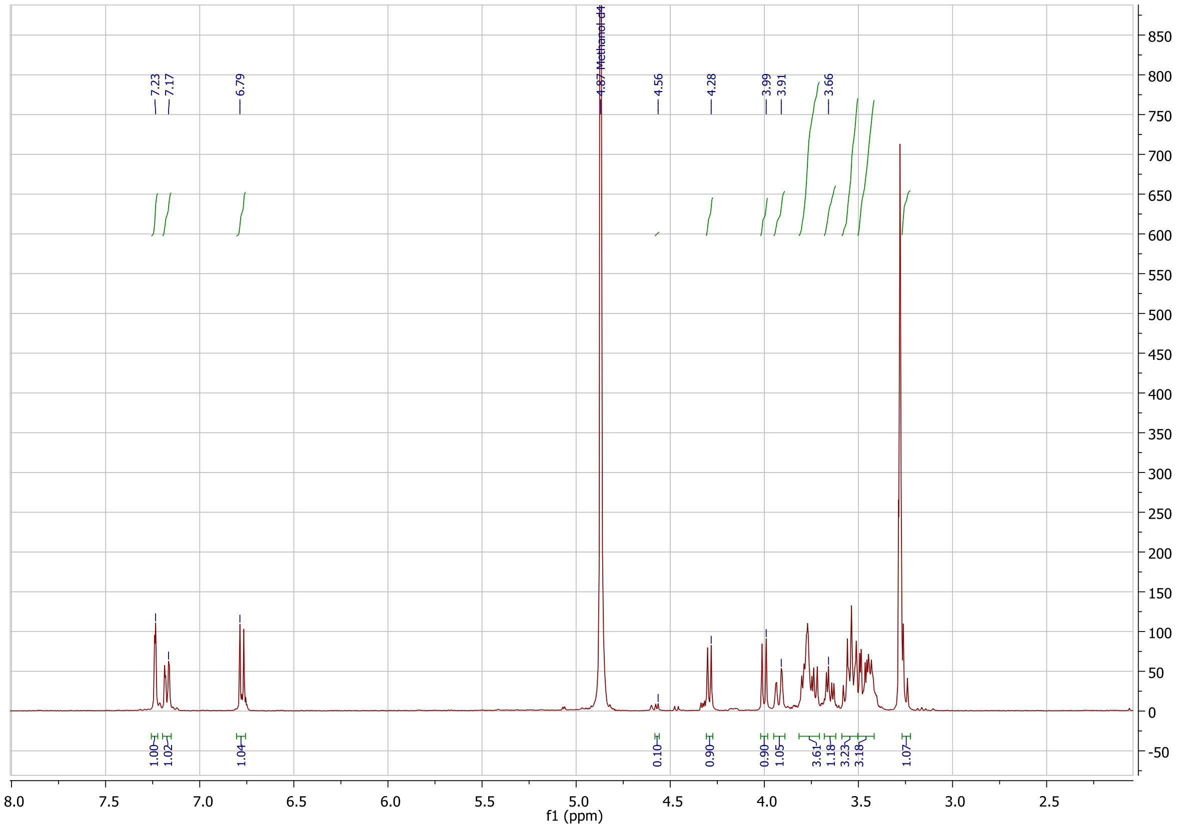

3.1.4 ^{13}C NMR spectrum for lactose adduct 3 (10:90 α:β, CD_3OD)

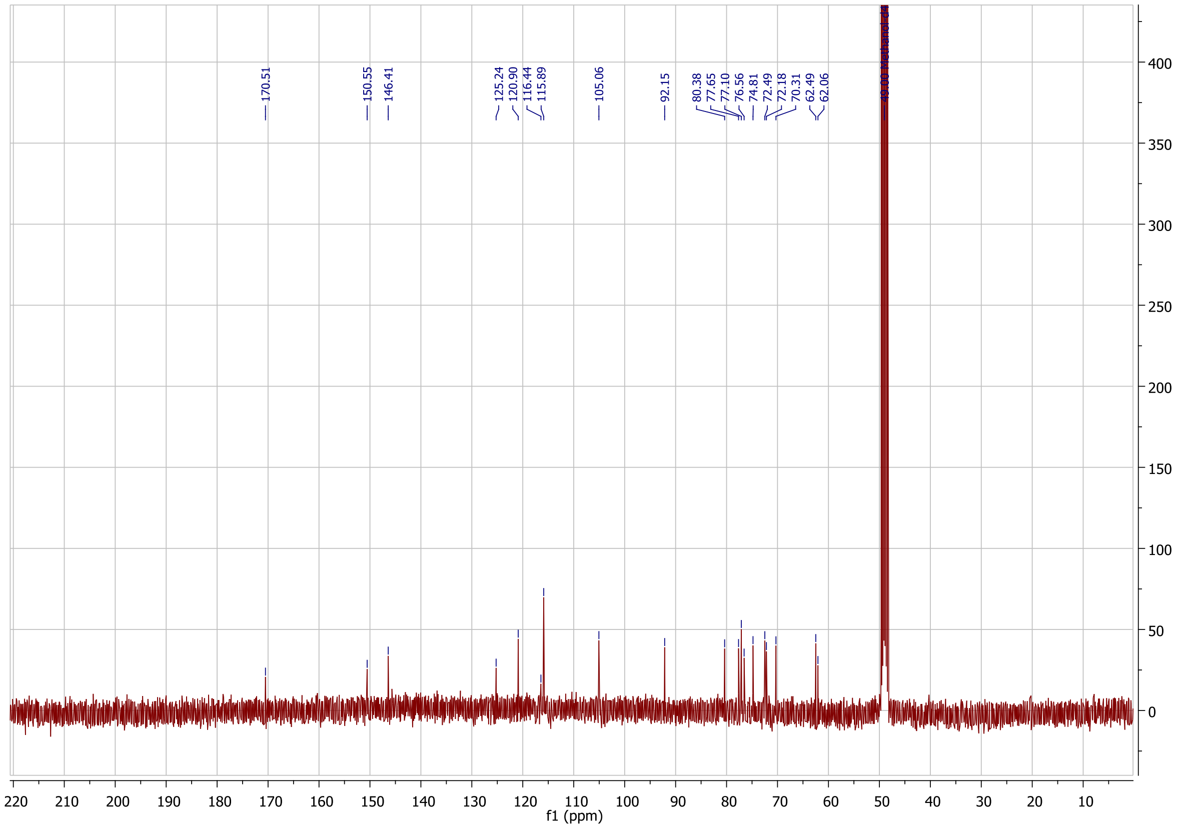

3.1.5 ^{1}H NMR spectrum for sialyllactose adduct 4 (9:91 α:β, CD_3OD)

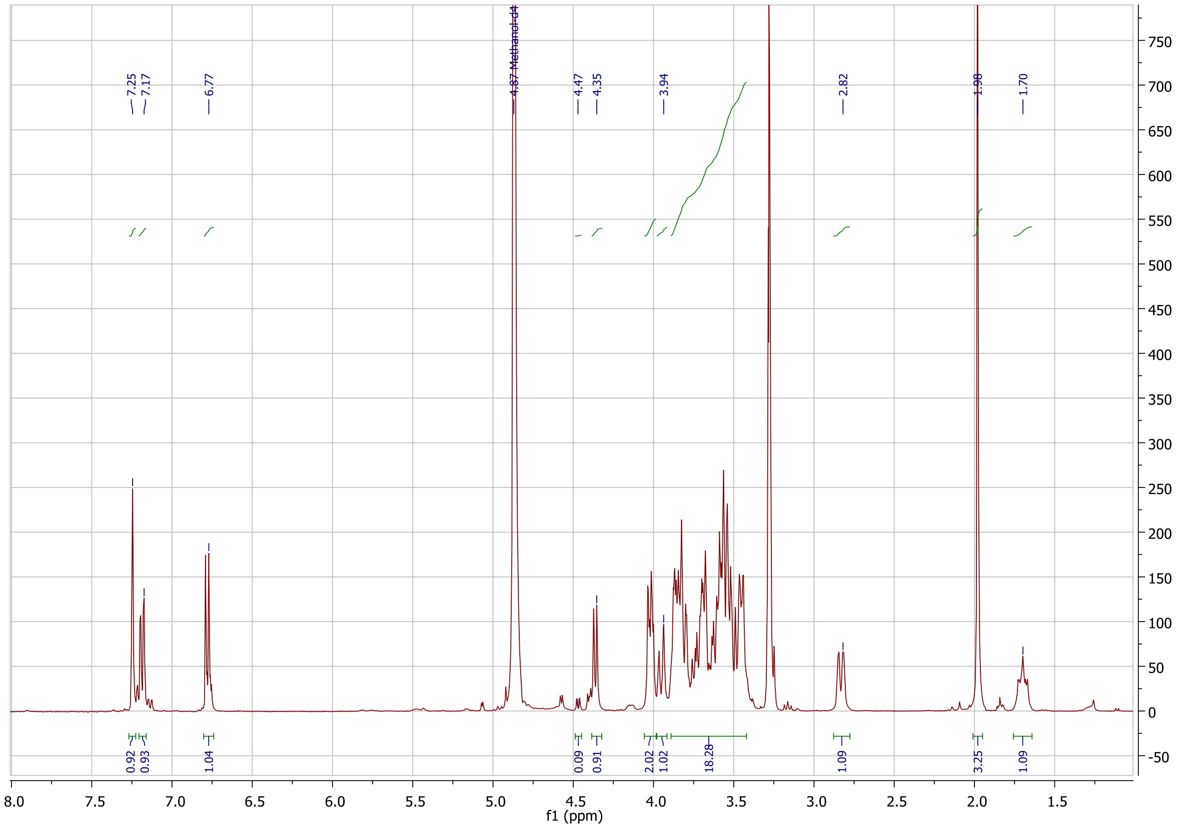

3.1.6 ^{13}C NMR spectrum for sialyllactose adduct 4 (9:91 α:β, CD_3OD)

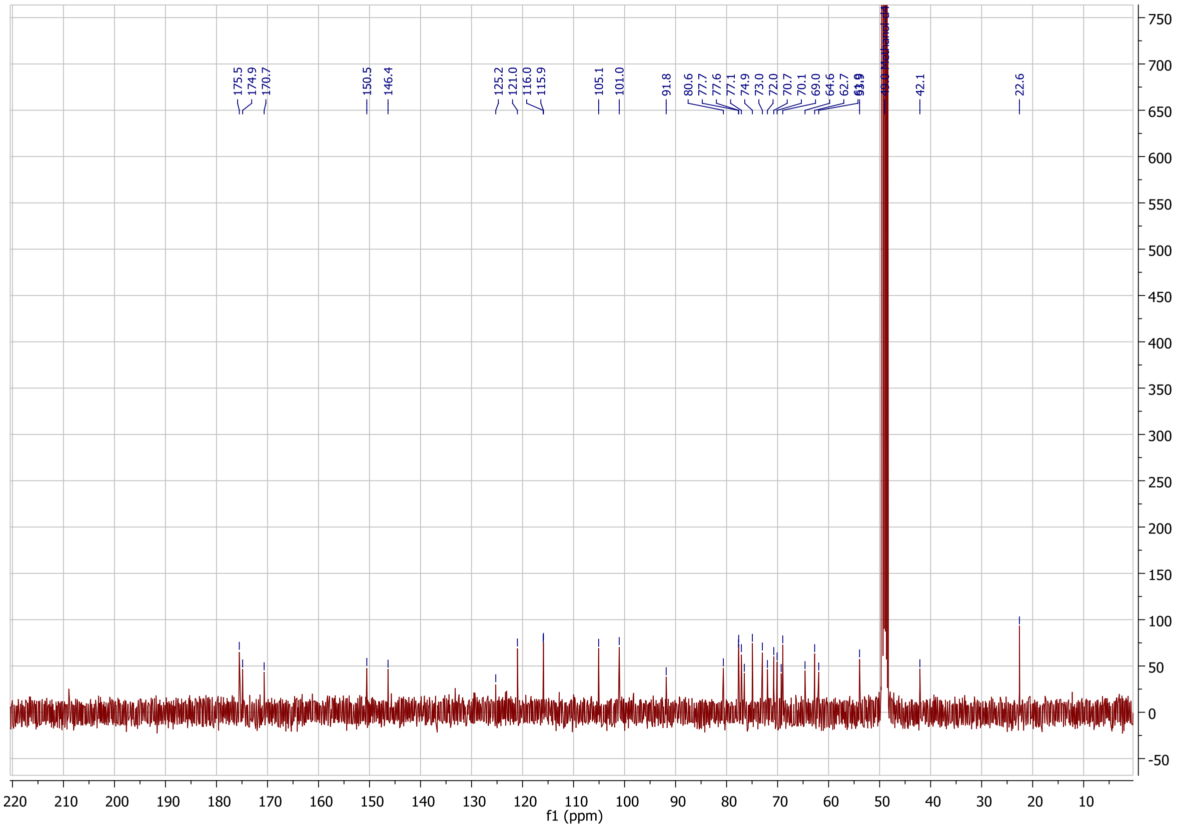

3.1.7 ^{1}H NMR spectrum for *N*-acetylglucosamine adduct 5 (14:86 α:β, CD_3OD)

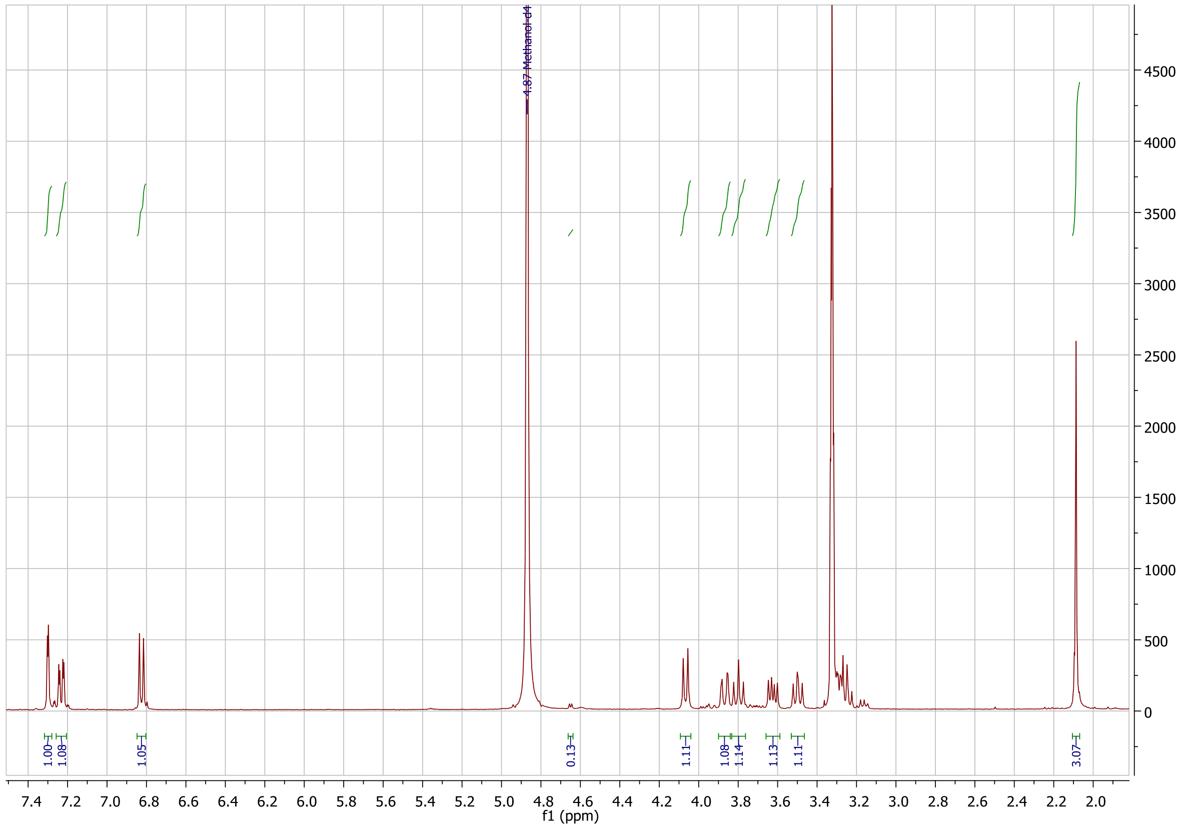

3.1.8 ^{13}C NMR spectrum for *N*-acetylglucosamine adduct 5 (14:86 α:β, CD_3OD)

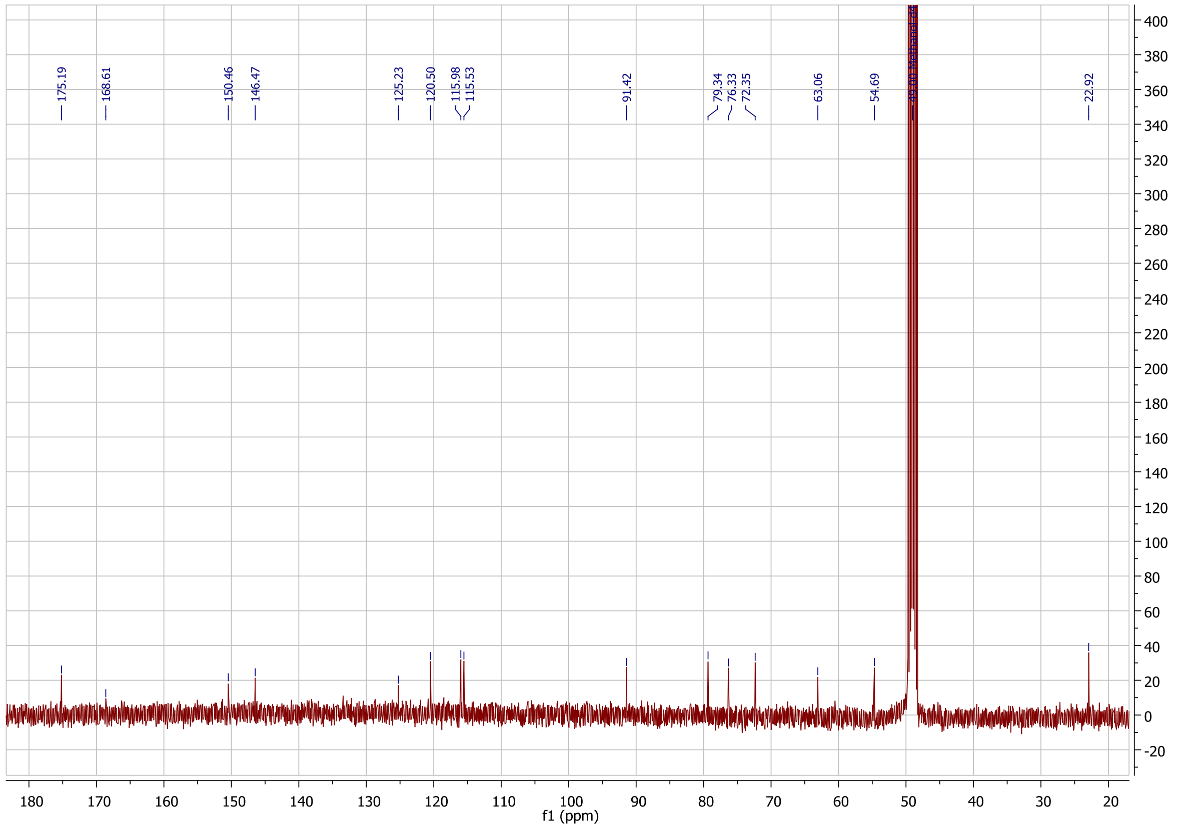

4. ^{1}H and ^{13}C NMR spectra of new compounds 6 -12

4.1.1 ^{1}H NMR spectrum for 2-deoxyglucose adduct 6 (22:78 α:β, CD_3OD)

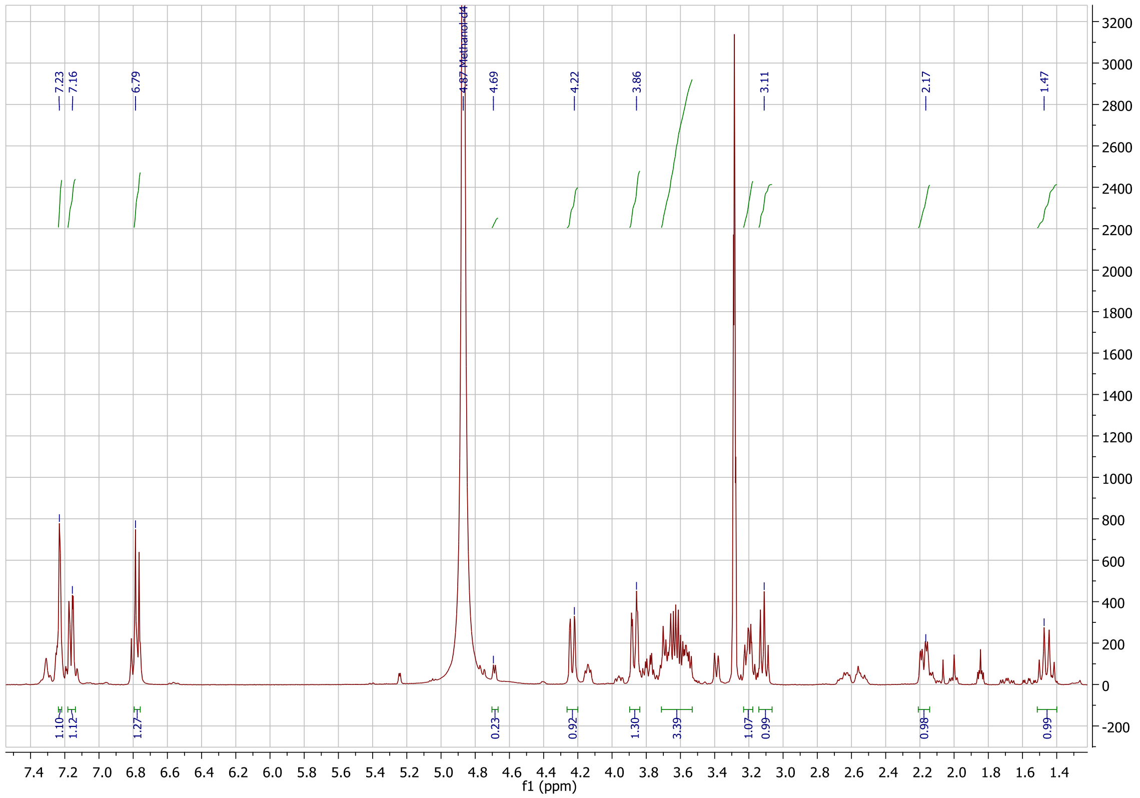

4.1.2 ^{13}C NMR spectrum for 2-deoxyglucose adduct 6 (22:78 α:β, CD_3OD)

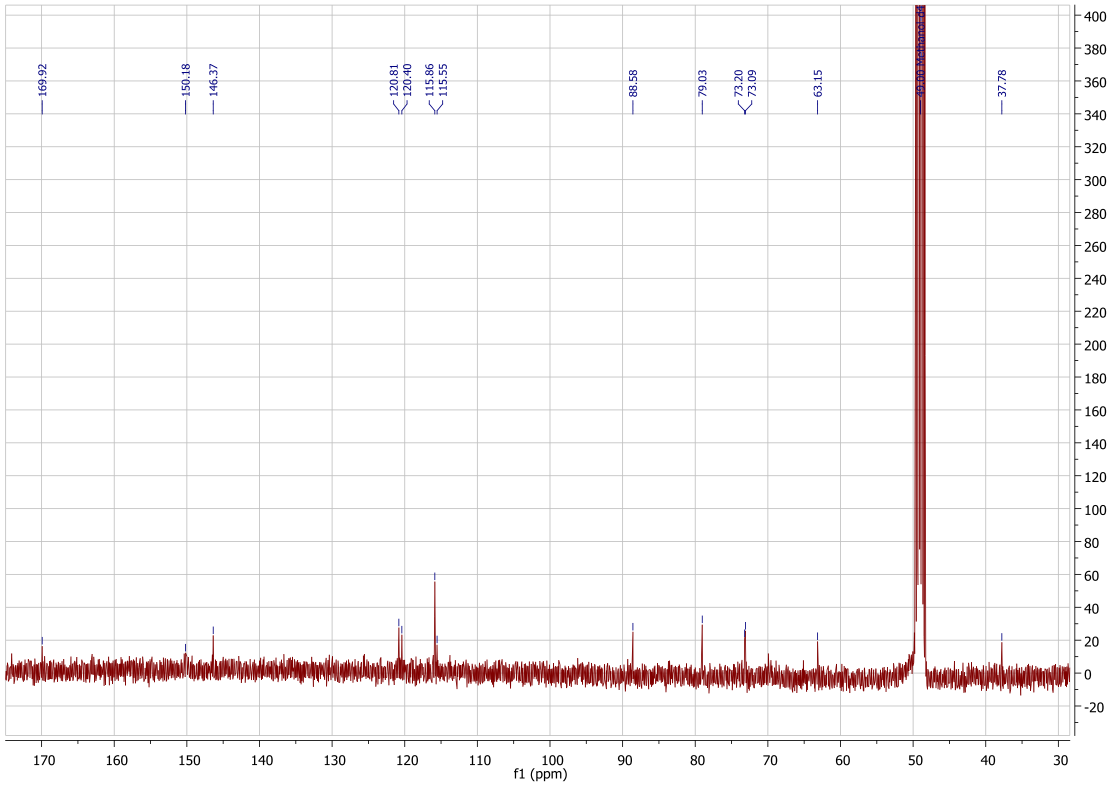

4.1.3 ^{1}H NMR spectrum for mannose adduct 7 (>99 % β, CD_3OD)

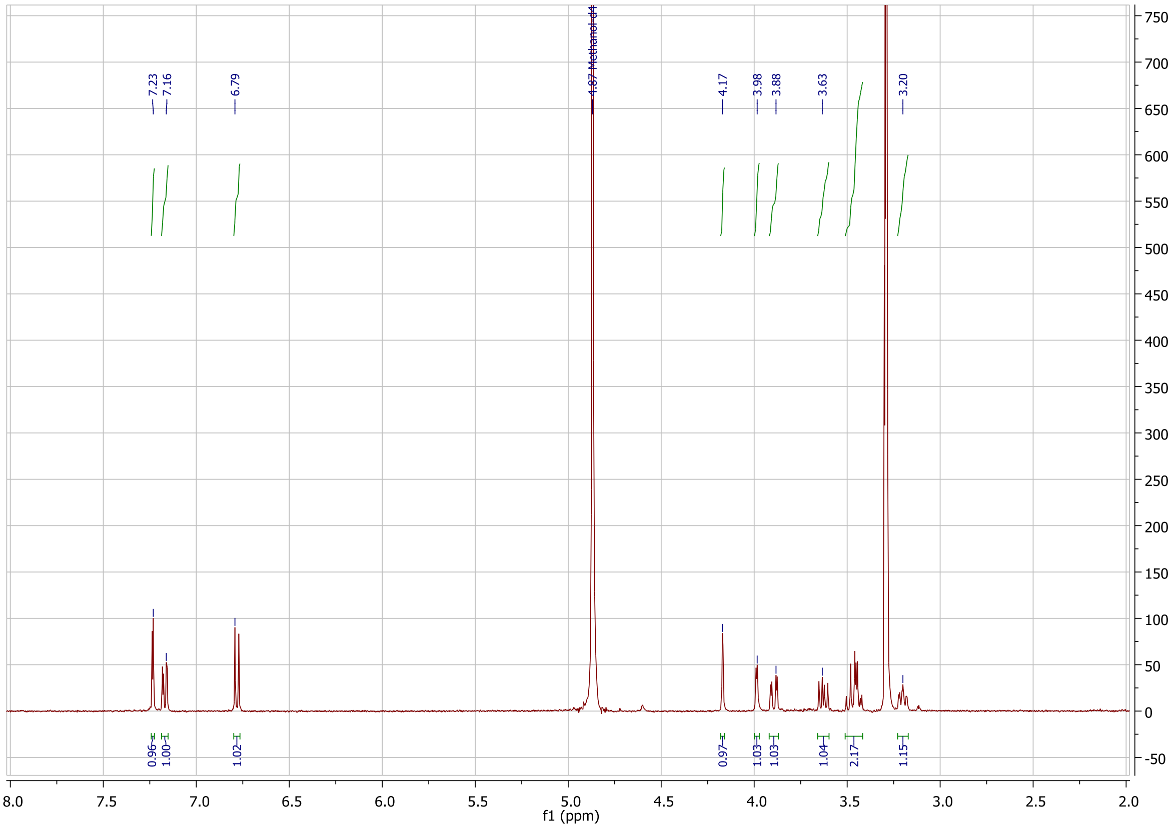

4.1.4 ^{13}C NMR spectrum for mannose adduct 7 (>99 % β, CD_3OD)

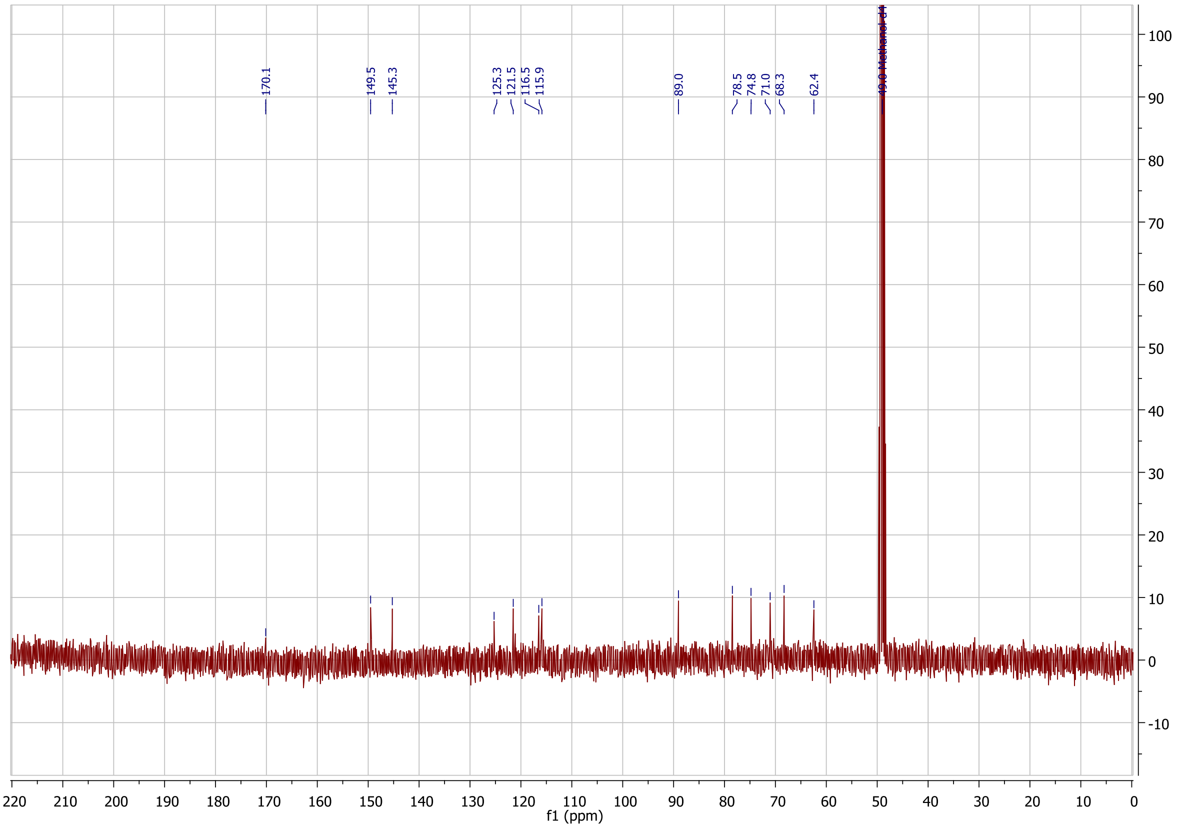

4.1.5 ^{1}H NMR spectrum for L-fucose adduct 8 (19:81 α:β, CD_3OD)

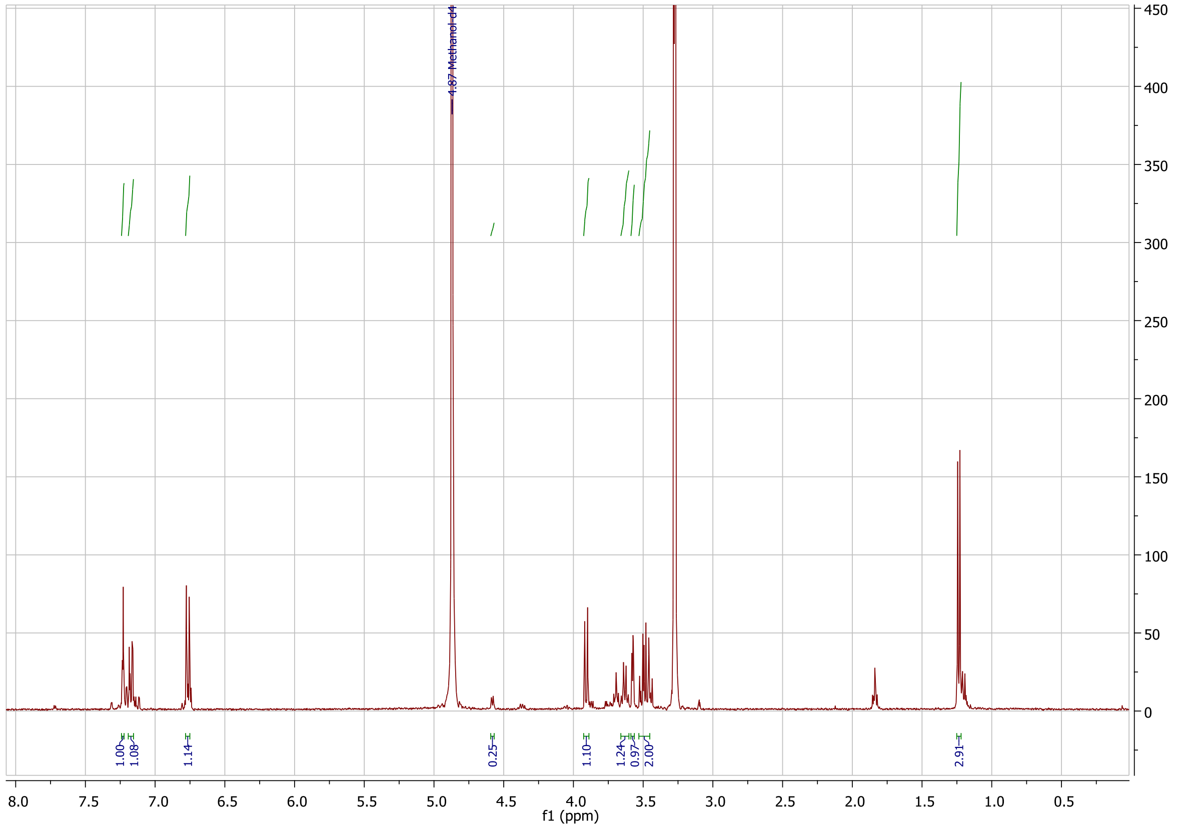

4.1.6 ^{13}C NMR spectrum for L-fucose adduct 8 (19:81 α:β, CD_3OD)

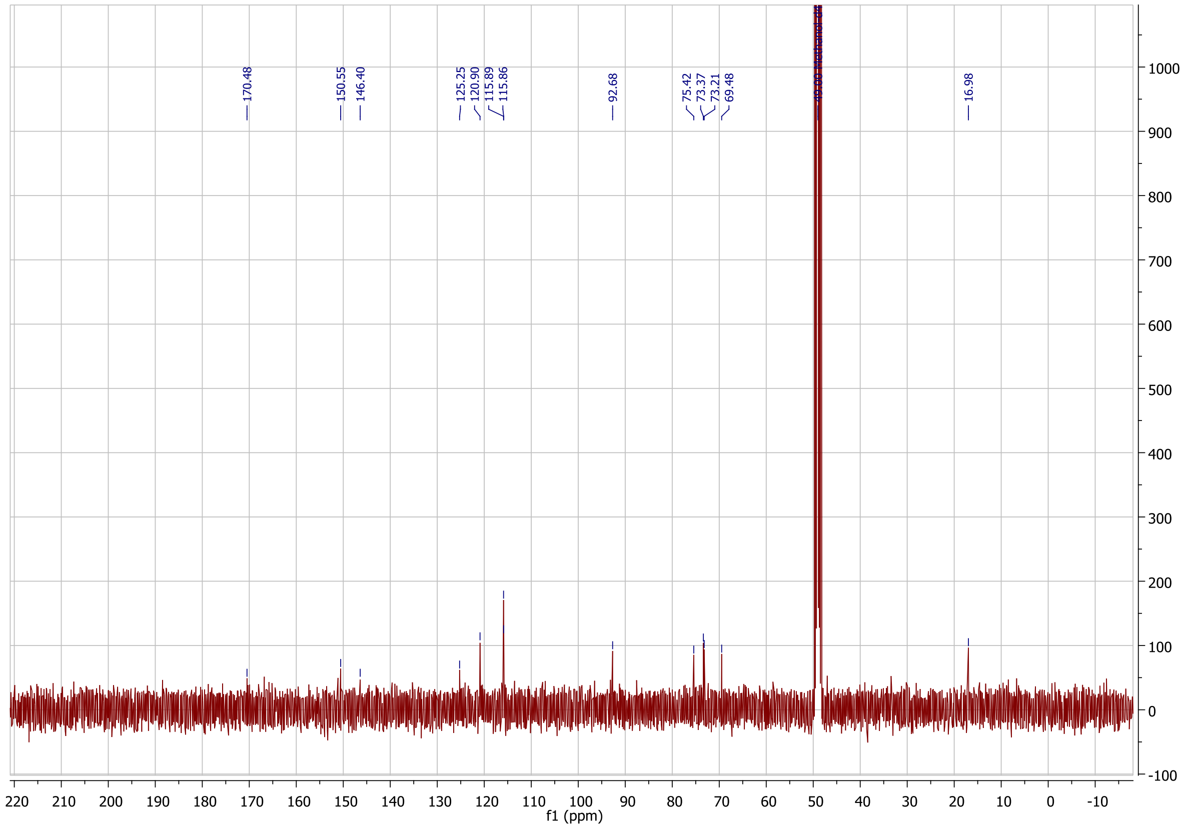

4.1.7 ^{1}H NMR spectrum for *N*-acetyllactosamine adduct 9 (10:90 α:β, CD_3OD)

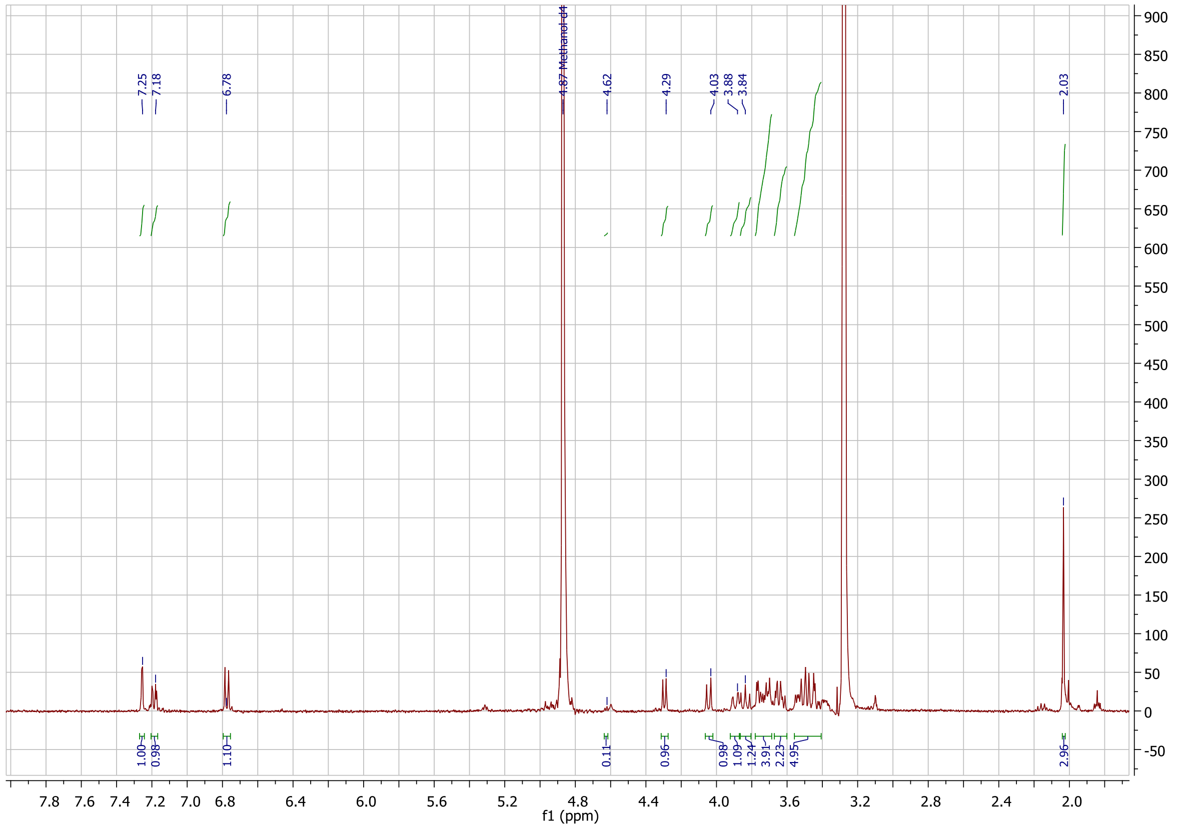

4.1.8 ^{13}C NMR spectrum for *N*-acetyllactosamine adduct 9 (10:90 α:β, CD_3OD)

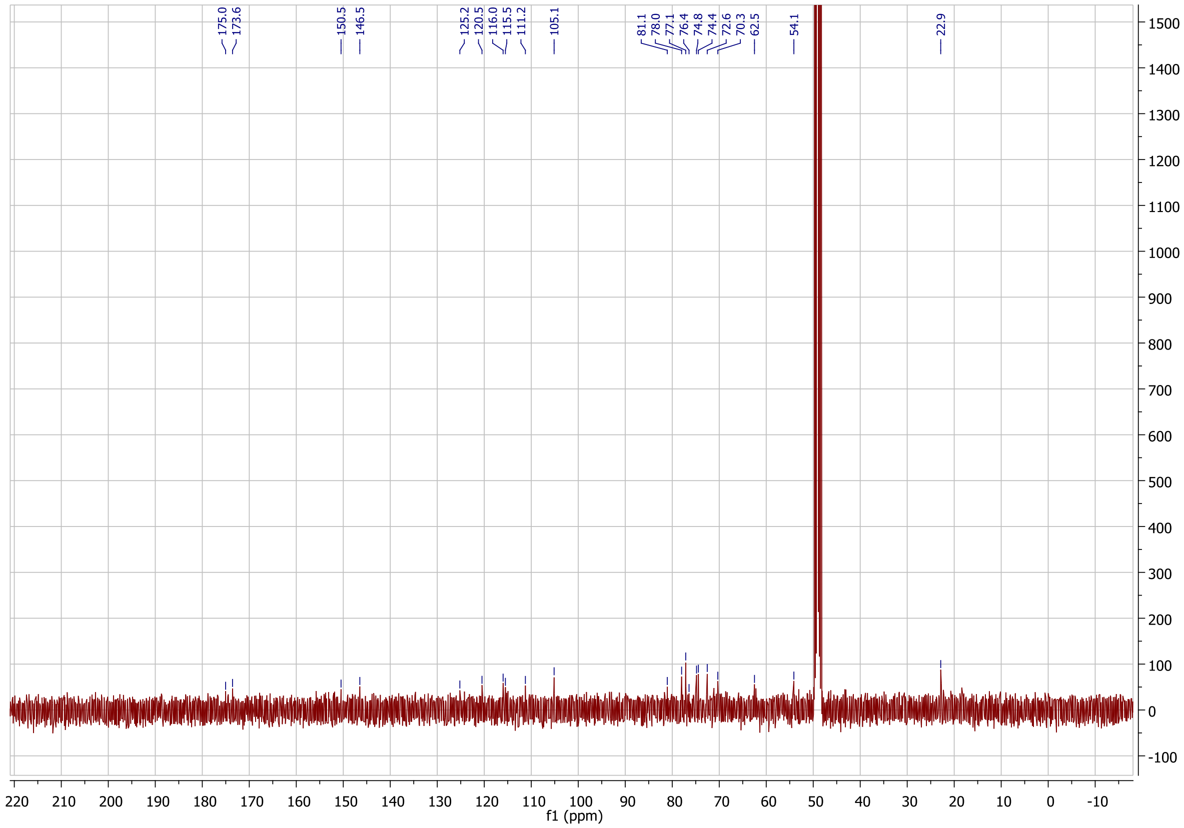

4.1.9 ^{1}H NMR spectrum for glucosamine adduct 10 (>99% β, CD_3OD)

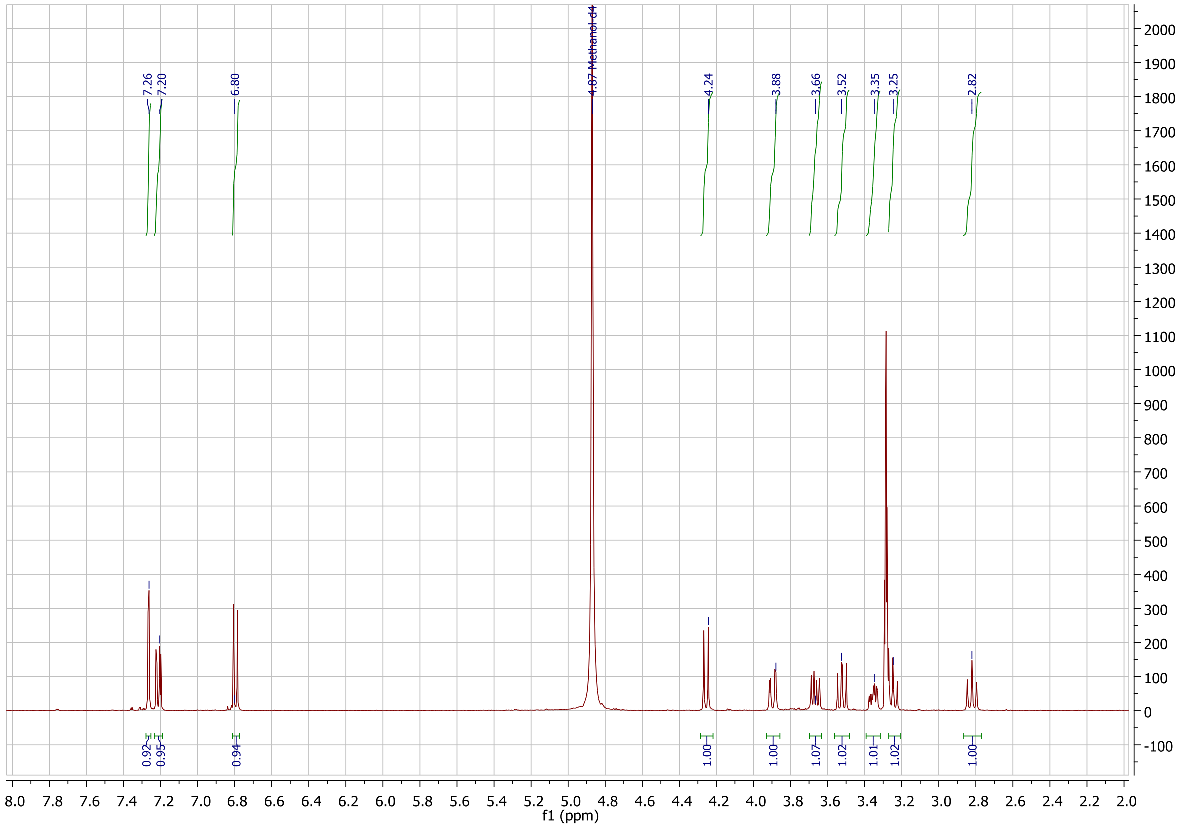

4.1.10 ^{13}C NMR spectrum for glucosamine adduct 10 (>99% β, CD_3OD)

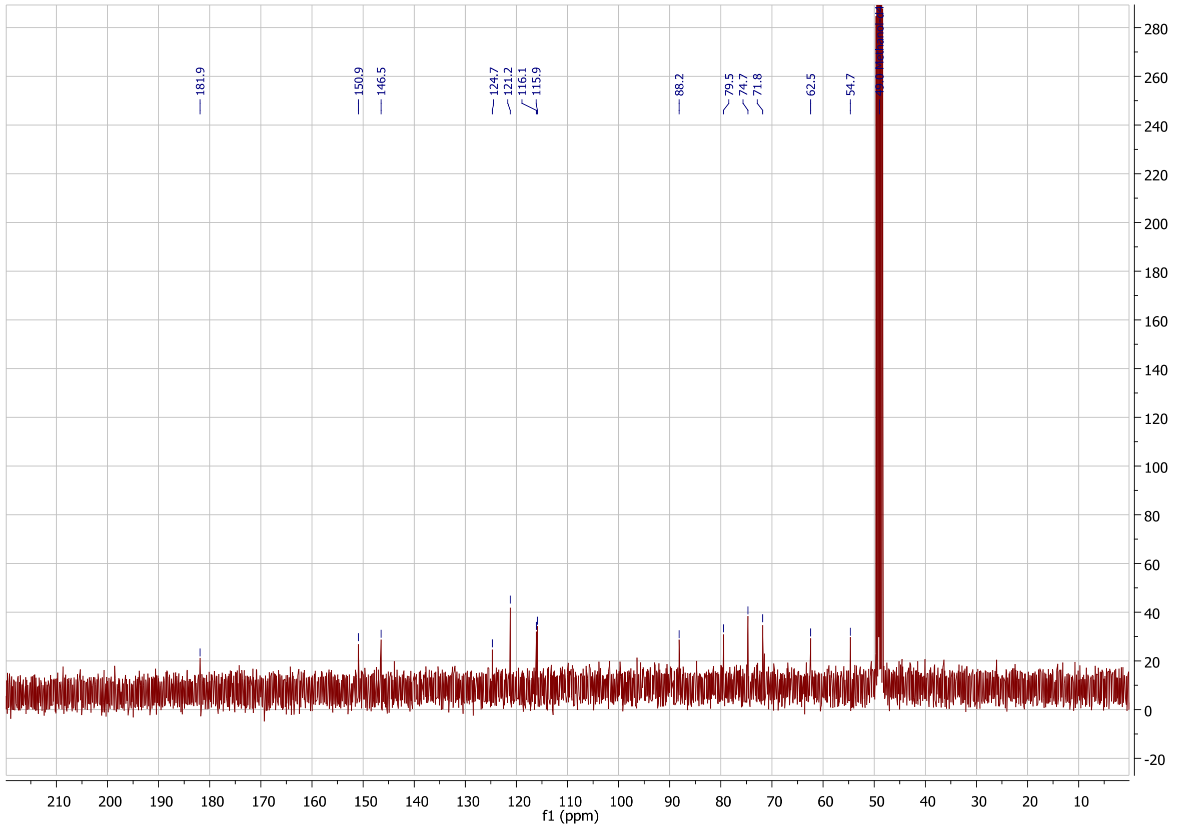

4.1.11 ^{1}H NMR spectrum for galactose adduct 11 (15:85 α:β, CD_3OD)

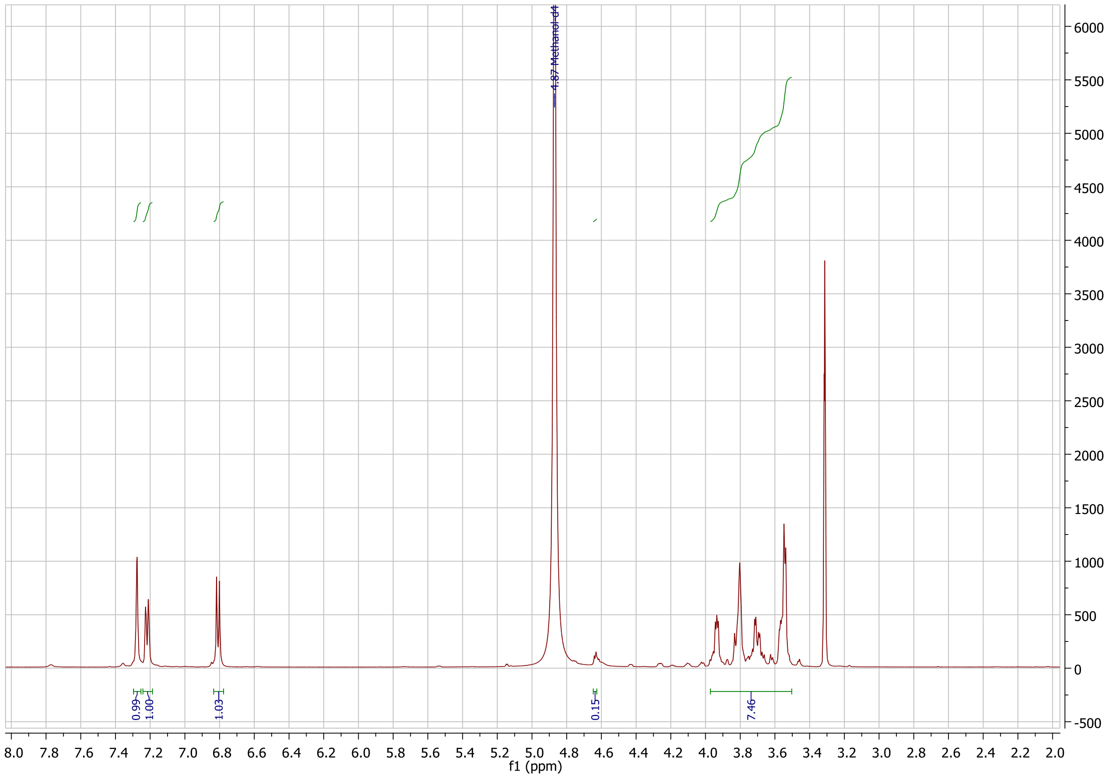

4.1.12 ^{13}C NMR spectrum for galactose adduct 11 (15:85 α:β, CD_3OD)

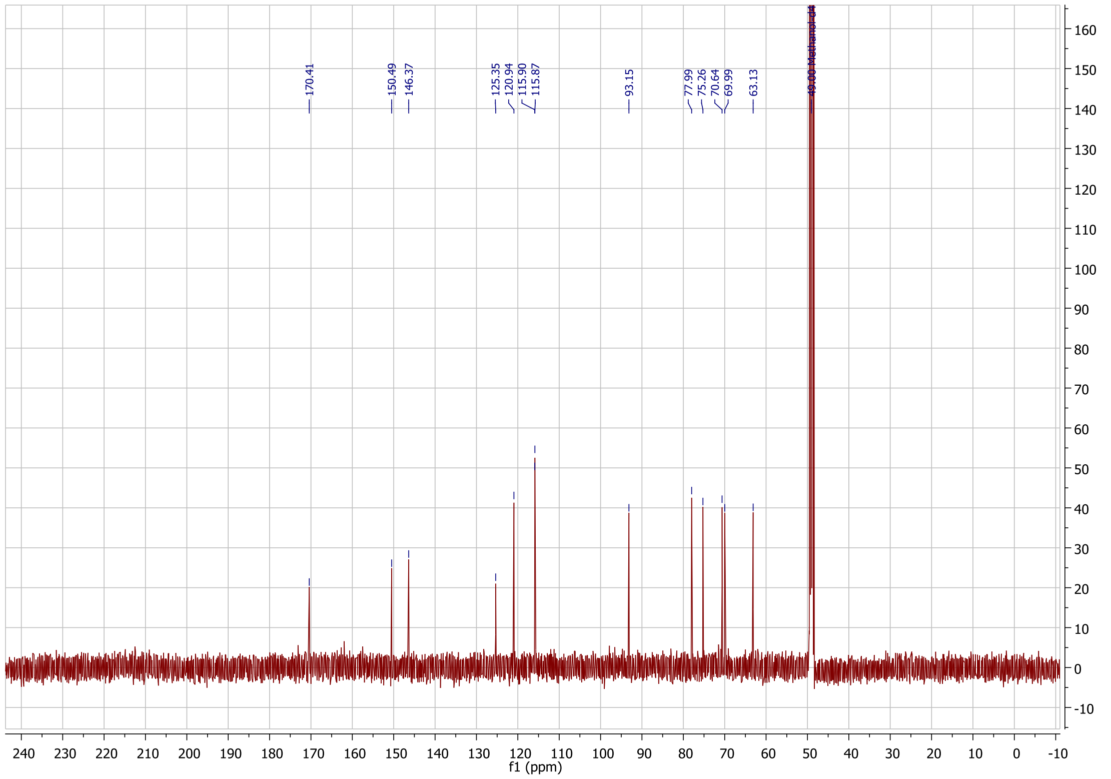

4.1.13 ^{1}H NMR spectrum for glucose-6-phosphate adduct 12 (>99% β, D_2O)

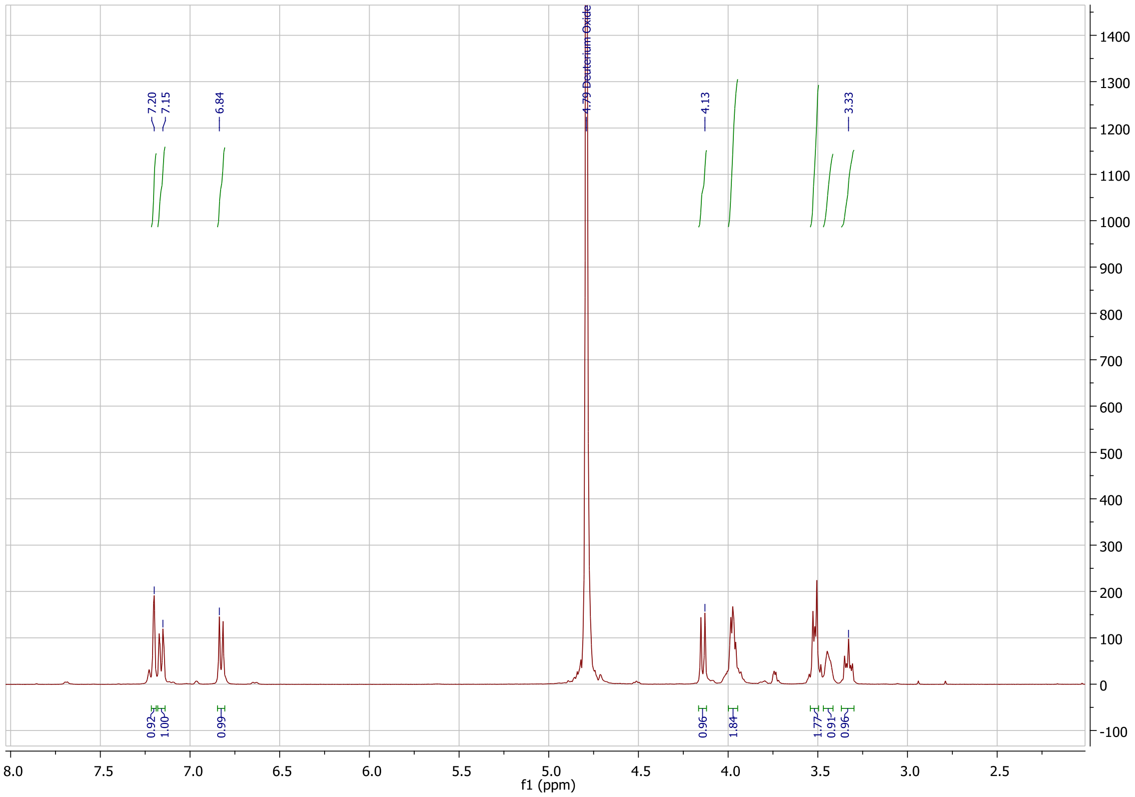

4.1.14 ^{13}C NMR spectrum for glucose-6-phosphate adduct 12 (>99% β, D_2O)

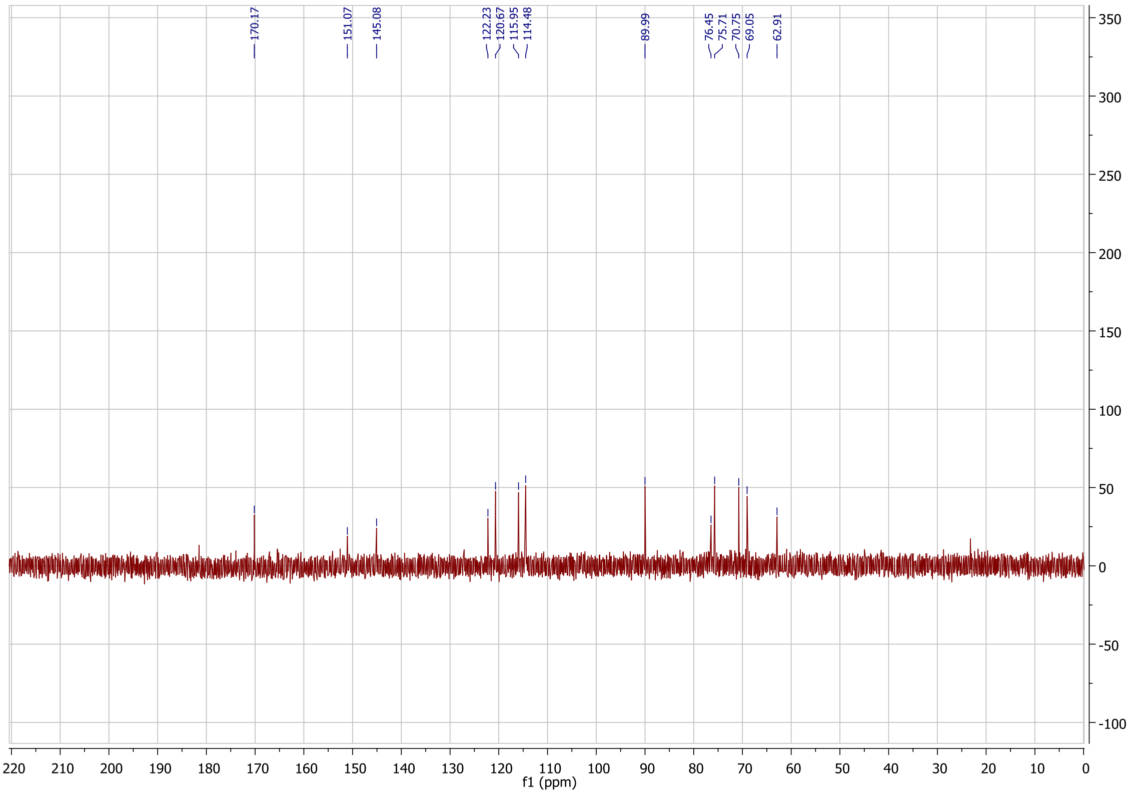

5. XRD for uncoated MNPs and MNPs coated with 2-8 and 10-12.

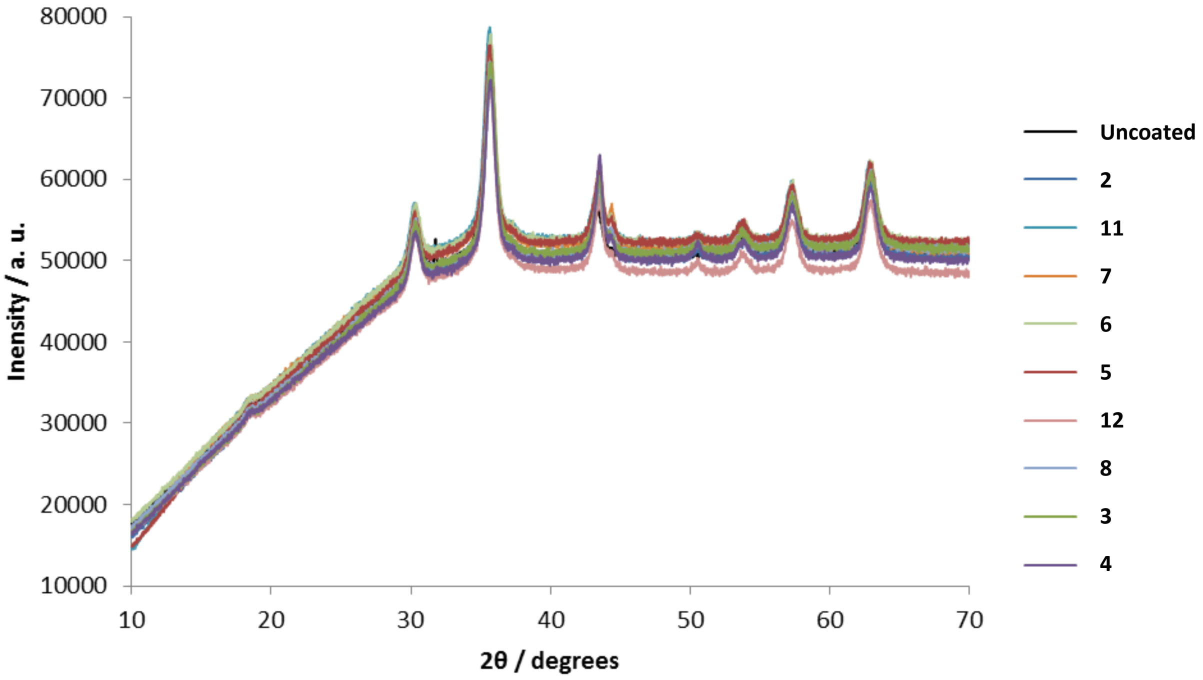

Figure S5.1: XRD spectral lines of uncoated MNPs, **2**-MNPs to **8**-MNPs and **10**-MNPs to **12**-MNPs.

Samples **2**-**8**-MNP and **10-12**-MNPs, as well as uncoated MNPs, were characterised by XRD. These XRD spectra are all very similar, consistent with the X-rays interacting weakly with the organic coatings and more strongly with the iron oxide surface. The recorded spectra gave peaks at scattering angles of 18.60, 30.30, 35.73, 43.50, 53.70, 57.32, and 63.02 degrees (Figure S5.1), which match with the (111), (220), (311), (400), (422), (511), (440) diffraction peaks of magnetite.[4]

6. TEM measurements of uncoated MNP size distribution

The diameters of the synthesized nanoparticles was measured from TEM images using imageJ software (n = 100). The sizes were grouped in intervals of 2 nm, and the number of particles in each group was counted (Figure S6.1). The mean diameter was calculated as 8.3 nm, with a standard deviation of 2.4 nm.

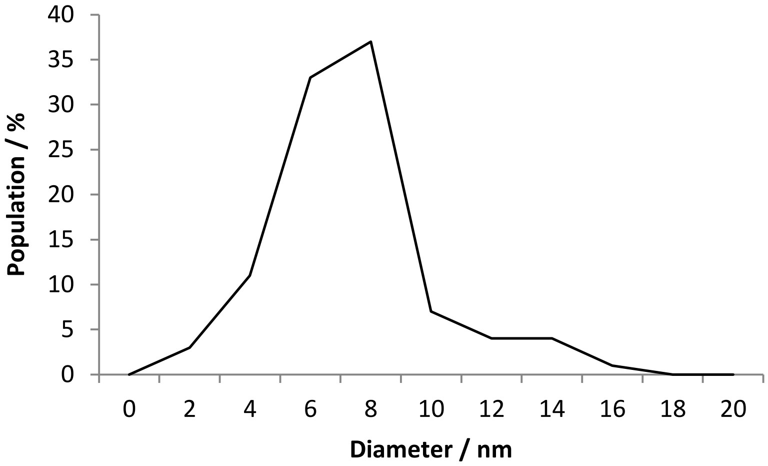

Figure S6.1: Size distribution graph of MNPs imaged by TEM (n = 100)

7. Inductively Coupled Plasma Atomic Emission Spectroscopy

ICP-AES measurements were obtained with the assistance of Mr. Paul Lythgoe, School of Earth & Environmental Sciences, University of Manchester.

Inductively Coupled Plasma Atomic Emission Spectroscopy (ICP-AES) was used to determine the presence and concentration of the saccharide-catechol adducts on the coated MNPs. As carbon, oxygen, nitrogen and hydrogen cannot be accurately measured by this technique, **12**-coated MNPs were investigated as phosphorus is suitable for ICP-AES analysis.

A sample of **12**-MNPs were prepared by dissolving completely in hydrochloric acid (15% w/v) at a concentration of 1 mg/mL and measured by ICP-AES.

The reported concentration of phosphorus was 16.41 mg/L or 0.01641 mg/mL. Phosphorus therefore accounted for 1.64% of **12**-MNP. Phosphorus (MW = 31 g/mol) was calculated to be 6.83% of the mass of coating molecule **12**, consequently molecule **12** ($C_{13}H_{17}N_2O_{11}PNa_2$, MW = 454 g/mol) is 24.0% of the weight of **12**-MNP. These magnetite nanoparticles have a density of 5.15 g/cm^3 and a radius of 4.16 nm (see Section 4.3) therefore weigh 1.55×10^{-18} g each. The amount of coating present on **12**-MNP is 4.8×10^{-19} g/particle or 1.06×10^{-21} mol/particle. If coating molecule **2** is assumed to have a rotation radius on the nanoparticle of 2.5×10^{-10} m then it will occupy 2×10^{-19} m^2/molecule or 1.2×10^5 m^2/mol. Molecule **12** therefore covers 1.27×10^{-16} m^2 of the surface of each nanoparticle. MNPs with radius 4.16 nm have a surface area of 2.17×10^{-16} m^2 therefore the coating molecule covers around 59% of the available surface area of each particle, a value similar to previous estimations.[5]

8. Estimation of the concentration of GlcNAc on 5-MNPs

Since **12**-coated MNPs had 0.0164 g phosphorus per gram of **12**-coated MNPs and 0.76 g Fe_3O_4 per gram of **12**-coated MNPs, there are 0.53×10^{-3} mol of **12** and 3.3×10^{-3} mol of Fe_3O_4 (9.8×10^{-3} mol of Fe) per gram of **12**-coated MNPs. This gives a 5.4% mol/mol ratio of **12** to Fe.

Assuming the extent of coating is the same for all saccharide-catechol adducts, **5**-coated MNPs at 0.25 mM total [Fe] would have 13 µM of the saccharide displayed on the surface.

9. Additional MRI relaxation data

9.1 Without WGA

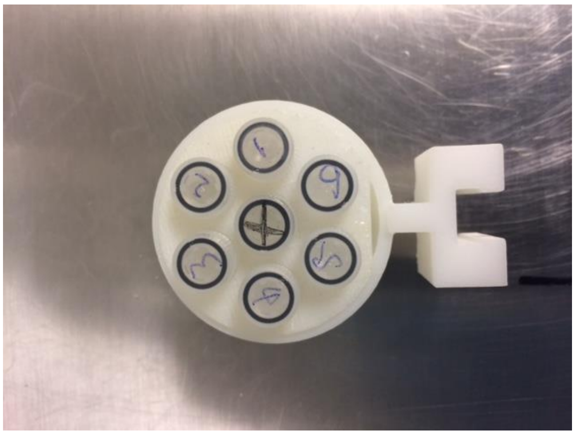

Figure S9.1: Image of MRI samples 1-6 in holder

Sample #	T_2 (ms)	T_1 (ms)
1 ([Fe] = 0.25 mM)	41 ± 1	2684 ± 86
2 ([Fe] = 0.1 mM)	67 ± 1	2921 ± 80
3 ([Fe] = 0.05 mM)	91 ± 1	2975 ± 70
4 ([Fe] = 0.025 mM)	102.4 ± 0.3	2929 ± 63
5 ([Fe] = 0.005 mM)	115.9 ± 0.2	3182 ± 77
6 ([Fe] = 0 mM)	122.5 ± 0.2	3202+/-84
X (centre, 0.2% magnevist in saline)	146.7 ± 0.1	241 ± 5

Table S1: MRI relaxation data for GlcNAc-coated MNP samples without WGA.

9.2 With WGA

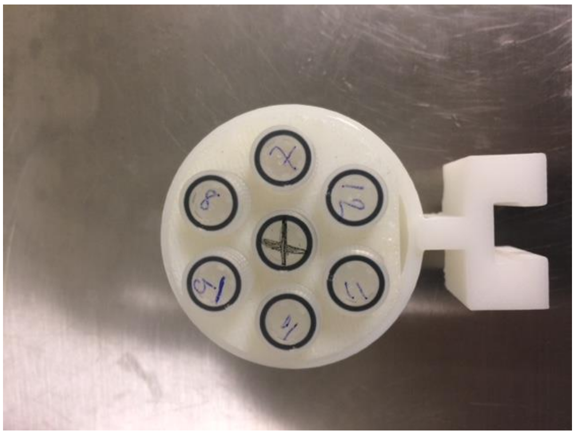

Figure S9.2: Image of MRI samples 7-12 in holder

Sample #	T_2 (ms)	T_1 (ms)
7 ([Fe] = 0.25 mM)	42 ± 2	2791 ± 106
8 ([Fe] = 0.1 mM)	70 ± 1	2984 ± 81
9 ([Fe] = 0.05 mM)	90 ± 1	3022 ± 72
10 ([Fe] = 0.025 mM)	101.6 ± 0.3	2973 ± 63
11 ([Fe] = 0.005 mM)	115.6 ± 0.4	3276 ± 89
12 ([Fe] = 0 mM)	121.4 ± 0.2	3274 ± 84
X (centre, 0.2% magnevist in saline)	147.3 ± 0.1	245 ± 5

Table S2: MRI relaxation data for GlcNAc-coated MNP samples with WGA.

9.3 Plot of iron concentration against 1/T_1 for GlcNAc-coated MNPs

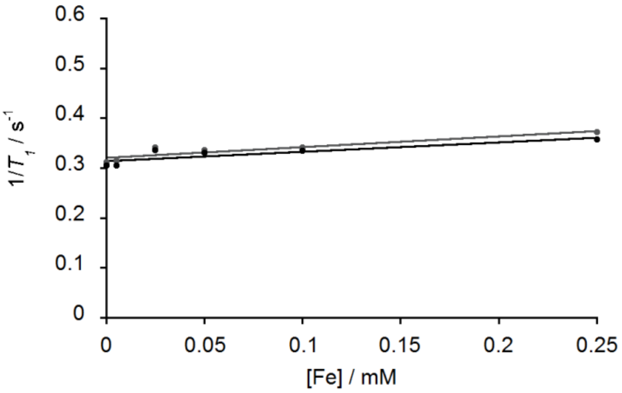

Figure S9.3: Plot of iron concentration against 1/T_1 for GlcNAc-coated MNPs with (black squares) and without WGA (grey circles).

10. Dynamic light scattering and zeta potential measurements

DLS and zeta potential measurements were carried out using a Malvern Zetasizer Nano ZSP 633 nm laser at 25 °C. Coated MNPs were suspended in PBS (pH 7.4), Milli-Q water or HEPES buffer (20 mM, pH 7.5 with 150 mM NaCl and 2 mM $CaCl_2$) at concentrations of 6 × 10^{-7} mg/mL. Particles were suspended by sonication with a probe-type sonicator for 10 mins followed by filtration using a Minisart® syringe filter of 200 µm. Measurements were proceed in folded capillary zeta cells with a scattering angle of 13° or 173°. The attenuation factor was 1.00 for all DLS samples.

DLS parameters: Material Fe_3O_4, refractive index 2.420, absorption 0.00. Dispersant: water, 25 °C, viscosity 0.8872 cP, refractive index 1.330. Equalisation time: 0 s. Each measurement was 10 runs, 1 s each. Analysis model: general purpose.

Zeta potential parameters: Material Fe_3O_4, refractive index 2.420, absorption 0.00. Dispersant: water, 25 °C, viscosity 0.8872 cP, refractive index 1.330, dielectric constant 78.5. $F_{(Ka)}$ model: Smoluchowski. $F_{(Ka)}$ value: 1.50. Each sample 2 measurements, 20 to 30 runs, 1 s each. Data processing: Auto mode. Mean count rate during measurements in Milli-Q water: uncoated MNPs, 133.0 Kcps; **1**-coated MNPs, 94.7 Kcps; **4**-coated MNPs, 79.3 Kcps; **9**-coated MNPs, 148.7 Kcps.

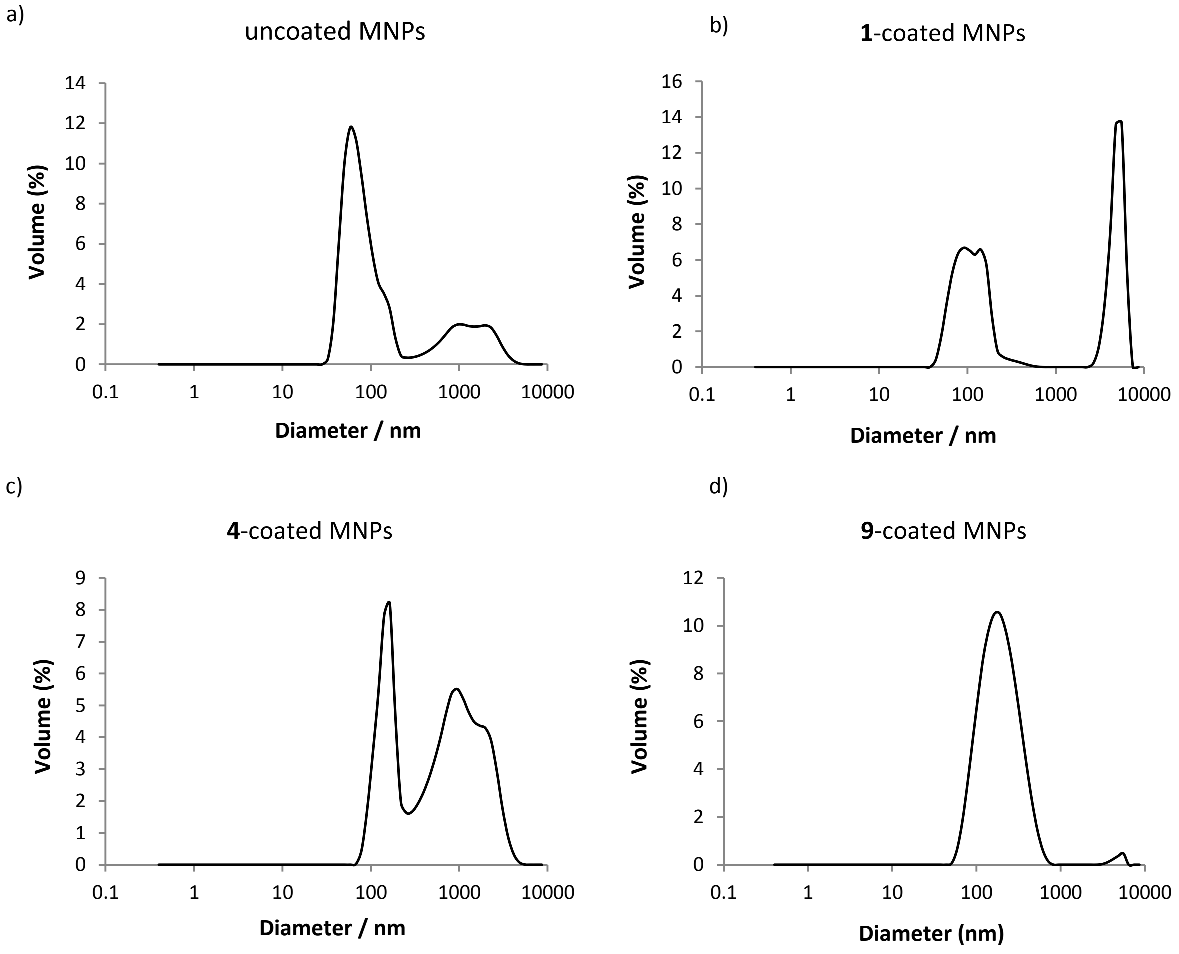

Figure S10.1: Plots showing DLS size distributions for (a) uncoated MNPs, (b) **1**-coated MNPs, (c) **4**-coated MNPs and (d) **9**-coated MNPs. MNPs in Milli-Q water at 6 × 10^{-7} mg/mL. Recorded at a scatter angle of 173°.

uncoated MNPs		
**Z-average**	**PdI**	
288.3	0.591	
	average	sd
**Peak 1**	81.18	37.62
**Peak 2**	901.3	355.4
**Peak 3**	2209	649.6

**1**-coated MNPs		
**Z-average**	**PdI**	
437	0.457	
	average	sd
**Peak 1**	88.01	22.39
**Peak 2**	165.3	59.1
**Peak 3**	4955	866.5

**4**-coated MNPs		
**Z-average**	**PdI**	
380.8	0.36	
	average	sd
**Peak 1**	152.8	39.91
**Peak 2**	1250	802.6

**9**-coated MNPs		
**Z-average**	**PdI**	
167.4	0.22	
	average	sd
**Peak 1**	120.1	63.48
**Peak 2**	4984	850.9

Figure S10.2: Mean z-average diameter (or radius) and mean polydispersity index in the DLS data for uncoated MNPs, **1**-coated MNPs and **4**-coated MNPs in Milli-Q water at 6×10^{-7} mg/mL. Average sizes and standard deviation (s.d.) for each peak. Recorded at a scatter angle of 173°.

a)

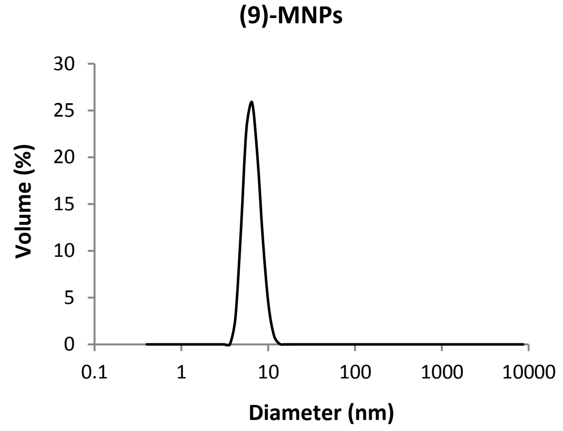

b)

**9**-coated MNPs		
**Z-average**	**PdI**	
55.86	1	
	average	s.d.
**Peak 1**	6.686	1.485

Figure S10.3: (a) Plot showing DLS size distribution for **9**-coated MNPs recorded at a scatter angle of 13°, soon after sonication. MNPs in Milli-Q water at 6×10^{-7} mg/mL. (b) Mean z-average diameter (or radius) and mean polydispersity index in these DLS data. Average size and standard deviation (s.d.) for peak.

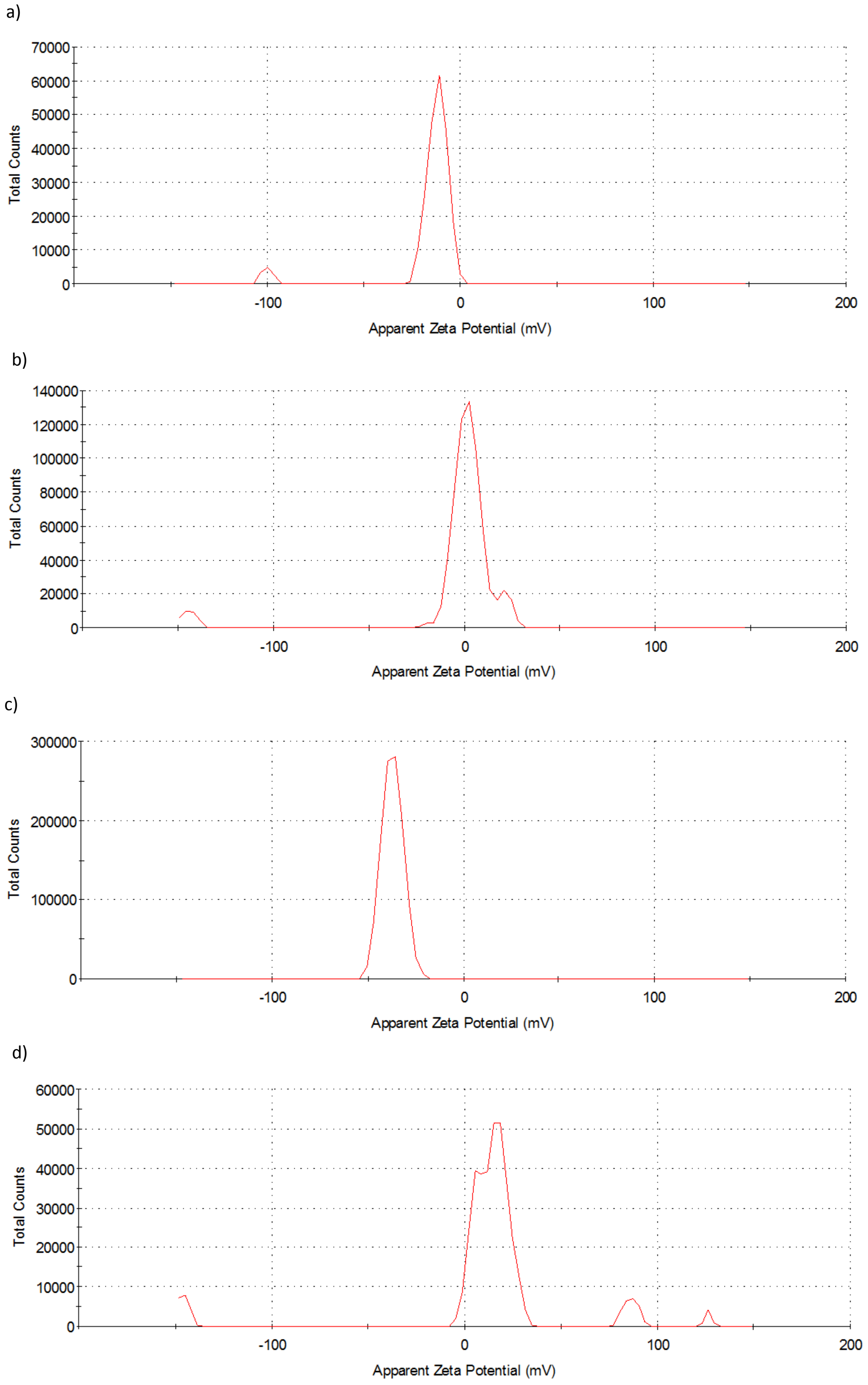

Figure S10.4: Representative plots showing zeta potentials for uncoated MNPs, **4**-coated MNPs, **9**-coated MNPs and **1**-coated MNPs in Milli-Q water at 6×10^{-7} mg/mL.

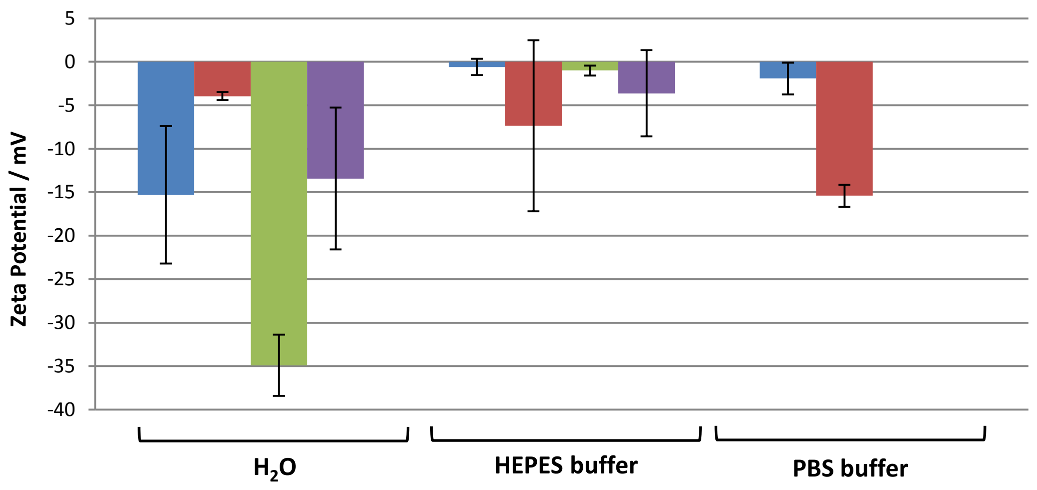

Figure S10.5: Average zeta potentials determined by DLS for uncoated MNPs (blue), **1**-coated MNPs (red), **4**-coated MNPs (green), and **9**-coated MNPs (purple) in Milli-Q water, in PBS (pH 7.4), or HEPES buffer (20 mM, pH 7.5 with 150 mM NaCl and 2 mM $CaCl_2$) at 6×10^{-7} mg/mL.

11. References

[1] M. Wendeler, L. Grinberg, X. Wang, P. E. Dawson and M. Baca, *Bioconjug. Chem*. 2014, **25**, 93-101.
[2] P. Crisalli and E. T. Kool, *J. Org. Chem*., 2013, **78**, 1184-1189.
[3] T. P. Coxon, T. W. Fallows, J. E. Gough and S. J. Webb, *Org. Biomol. Chem*. 2015, **13** 10751-10761.
[4] S. Adireddy, C. Lin, V. Palshin, Y. Dong, R. Cole and G. Caruntu, *J. Phys. Chem. C*, 2009, **113**, 20800-20811.
[5] F. de Cogan, A. Booth, J. E. Gough and S. J. Webb, *Angew. Chem. Int. Ed*., 2011, **50**, 12290-12293.
